# Nanoparticles Functionalized by Conducting Polymers and Their Electrorheological and Magnetorheological Applications

**DOI:** 10.3390/polym12010204

**Published:** 2020-01-13

**Authors:** Yu Zhen Dong, Kisuk Choi, Seung Hyuk Kwon, Jae-Do Nam, Hyoung Jin Choi

**Affiliations:** 1 Department of Polymer Science and Engineering, Inha University, Incheon 22212, Korea; 22152270@inha.edu (Y.Z.D.); focalis@naver.com (S.H.K.); 2 Department of Polymer Science and Engineering, Sungkyunkwan University, Suwon 16419, Korea; kisuk929@skku.edu (K.C.); jdnam@skku.edu (J.-D.N.)

**Keywords:** nanoparticle, conducting polymer, electrorheological, magnetorheological

## Abstract

Conducting polymer-coated nanoparticles used in electrorheological (ER) and magnetorheological (MR) fluids are reviewed along with their fabrication methods, morphologies, thermal properties, sedimentation stabilities, dielectric properties, and ER and MR characteristics under applied electric or magnetic fields. After functionalization of the conducting polymers, the nanoparticles exhibited properties suitable for use as ER materials, and materials in which magnetic particles are used as a core could also be applied as MR materials. The conducting polymers covered in this study included polyaniline and its derivatives, poly(3,4-ethylenedioxythiophene), poly(3-octylthiophene), polypyrrole, and poly(diphenylamine). The modified nanoparticles included polystyrene, poly(methyl methacrylate), silica, titanium dioxide, maghemite, magnetite, and nanoclay. This article reviews many core-shell structured conducting polymer-coated nanoparticles used in ER and MR fluids and is expected to contribute to the understanding and development of ER and MR materials.

## 1. Introduction

Smart materials, also called intelligent materials, can sense external stimuli, such as light, temperature, pH, stress, strain, chemical, nuclear radiation, electric fields, and magnetic fields. In particular, these types of materials can usually select and control the degree of response according to the design requirement, and can quickly go back to their initial phase when the external stimulus is eliminated [1,2,3]. Among various smart materials, the electric field-responsive smart particle suspension, called an electrorheological (ER) fluid, can change state from liquid-like to solid-like immediately and reversibly with an applied electrical field [4,5,6,7]. The suspended electro-responsive particles in a non-conducting medium align along the applied electrical field direction, resulting in the build-up of chain-like forms that increase the shear viscosity dramatically with the appearance of a yield stress [8]. Therefore, their mechanical and rheological properties can be changed considerably with an electrical field strength within milliseconds and return to an original state when the applied field is turned off [9,10,11]. Similar to an ER fluid, a magnetorheological (MR) suspension usually consists of magnetic particles suspended in a carrier liquid that converts rapidly to a solid-like form under a magnetic field and returns to a liquid-like phase when the applied field is withdrawn [12,13]. These fascinating ER and MR fluids have attracted significant attention in many industrial applications, such as damper, tactile display, material polishing, robotics, clutches, and microfluidics [14,15,16,17,18,19].

Various dry-based ER fluids include inorganic non-metallics of typical ionic crystalline materials and semi-conducting polymers possessing a π-conjugated form and electrical conductivity [20,21], displaying unusual properties such as large electron affinity and low ionization potential. Local electron distribution of the dispersed particles causes their ER behavior with an electrical field. Especially, several conducting polymeric particles have been introduced as anhydrous ER particles to overcome the disadvantages from the wet-base ER materials, including a narrow operation temperature and device corrosion [22]. In addition, conducting polymers are great candidate ER materials owing to their reasonable density, excellent thermal and environmental stability, simple synthesis, and easy adjustment of the electrical conductivity. Thus far, conducting polymers, such as polyaniline (PANI), poly(diphenylamine), polypyrrole (PPy), poly(*p*-phenylene) (PPP), poly(3,4-ethylenedioxythiophene) (PEDOT), and their derivatives, have been introduced for use in ER fluids [23,24,25,26,27,28].

Along with chemical component of the ER particles, their morphological characteristics, such as size, shape, and size distribution, have attracted considerable attention [29]. In particular, core-shell shaped particles of an inorganic or a polymeric core and conducting shell become attractive candidates for introducing new mechanisms and materials in ER studies [30,31,32]. Compared to pristine conducting polymer-based ER fluids, conducting particles with a core-shell structure could be polarized shortly, which improves the polarization behavior of particles according to the electrical field [33]. The nanostructured functional coating of a thin conducting layer is one of the best ways of reducing the cost of conducting polymers at the same time improving the transparency of the particles, which is used widely in the photographic and display industry. In addition, the application of nano-sized cores may provide the composites special physical and chemical properties, especially such as optical characteristics [34].

With MR fluids, a range of magnetic particles, such as carbonyl iron (CI), nickel, Fe_3_O_4_, γ-Fe_2_O_3_, ZnFe_2_O_4_, and CoFe_2_O_4_, have been widely studied as MR materials [35,36,37,38,39]. Meanwhile, the sedimentation problem induced by the density mismatch between magnetic particles and the carrier liquid is one of the main problems limiting the application of MR fluids in industry. Considerable efforts have been made to solve this problem, such as combining magnetic materials with low-density materials to obtain magnetic composite materials, and employing additives in MR fluids [40,41]. In particular, when a polymer is used to form core-shell structured magnetic particle-polymer composites, the polymer shell can reduce the density difference between the magnetic materials and the carrier liquid to enhance the sedimentation stability of an MR fluid. In addition, it can protect the magnetic cores from corrosion and oxidation, thereby improving the stability of the MR properties [42,43].

**Scheme 1:**
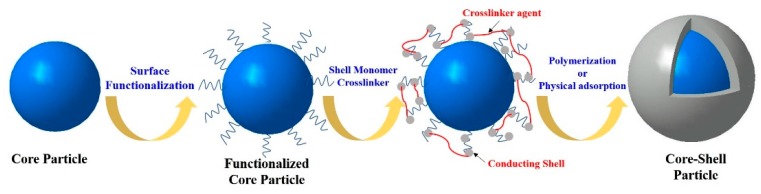
Schematic diagram of the fabrication method of conducting core-shell particles.

Core–shell particles can be fabricated using a multi-step process, including the synthesis of the core particles and a following coating step using conductive polymers as the electro-responsive shell, as shown in Scheme 1. Several polymerization techniques have been introduced to synthesize monodisperse polymeric cores, such as dispersion or seeded emulsion polymerization processes regarding the particle size or shape [44,45]. In addition, some inorganic materials with different morphologies, such as silica, titania, and iron oxide, are also used as the core. Special methods are then required to direct the coating technique onto core parts. Surfactants or grafting agents could help modifying the core for better chemical interactions or physical adsorption with the conductive polymer shell along with a controlled releasing technique [46,47]. In particular, when a conducting polymer coated core-shell structured composite material is used as the MR material, the materials used as the core need to be magnetic, and both soft magnetic carbonyl iron (CI) and Fe_3_O_4_ are the most competitive candidates. A series of polymer-coated core-shell structured composites with Fe_3_O_4_ as a core have been successfully synthesized, such as core-shell structured Fe_3_O_4_@Au/polydopamine synthesized by an in situ redox-oxidation polymerization [48], Fe_3_O_4_@polyaniline@Au synthesized by a ultrasound-assisted in situ surface polymerization [49] and polydopamine-sandwiched Au shell-coated Fe_3_O_4_ core via a simple oxidation polymerization [50]. Furthermore, conducting polymer coated core-shell structured composites with micro-sized CI particles as the core have been researched as MR materials, such as CI/polyindole (PIn) [51] and CI/PANI [52].

This paper reviews the synthesis of core-shell structured conducting polymer-coated nanoparticles used in ER and MR fluids, as well as their interesting smart rheological characteristics. Typical characterization, such as morphologies, thermal properties, sedimentation stabilities, and dielectric properties, are also included. The ER and MR performance of these materials are observed using steady shear and oscillation tests carried out by rotational rheometers.

## 2. Fabrication and Morphologies

### 2.1. PANI and Its Derivatives

#### 2.1.1. PANI

As one of the most common conductive polymers, PANI has become an important material in various fields owing to its excellent electrochemical performance, environmental stability, biocompatibility, and ease of synthesis [53,54,55]. In particular, the electrical conductivity of PANI can be adjusted conveniently by doping and de-doping with acid and base and has been studied extensively for use in ER fluids [56,57]. Meanwhile, the morphology of generally synthesized PANI tends to be irregular. Although many methods for the synthesis of fibrous or tubular PANIs have been reported including template synthesis [58], when a suitable material is adopted as a core to fabricate core-shell shaped ER materials, the core can act as a template to homogenize the morphology of the PANI, and suppress the electrical breakdown of the material under an electric field.

##### Polymeric Core

Among various conducting polymers, PANI and its derivatives have several advantages, such as easy fabrication, reasonable density, and controllable conductivity with a rather simple doping and de-doping method, and low production cost [59,60,61,62,63,64]. A series of PANI, its derivatives, and their organic or inorganic composite materials with different morphologies have been studied widely as ER materials.

**Scheme 2:**
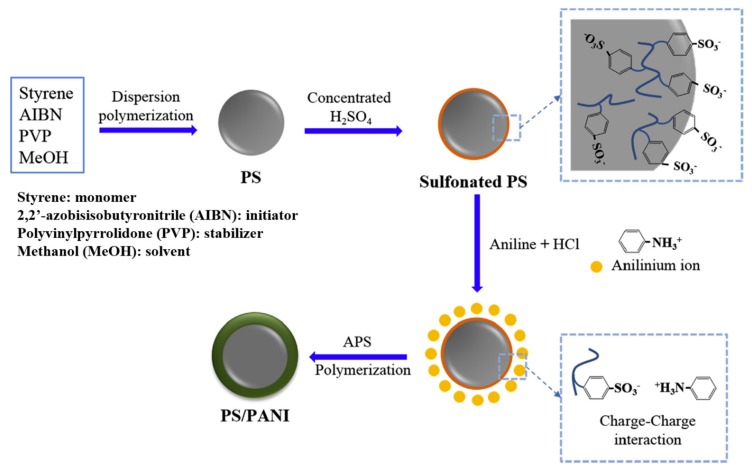
Schematic diagram of synthetic route of PS/PANI nanoparticles (Piao et al. [61], © Elsevier, 2017).

**Figure 1:**
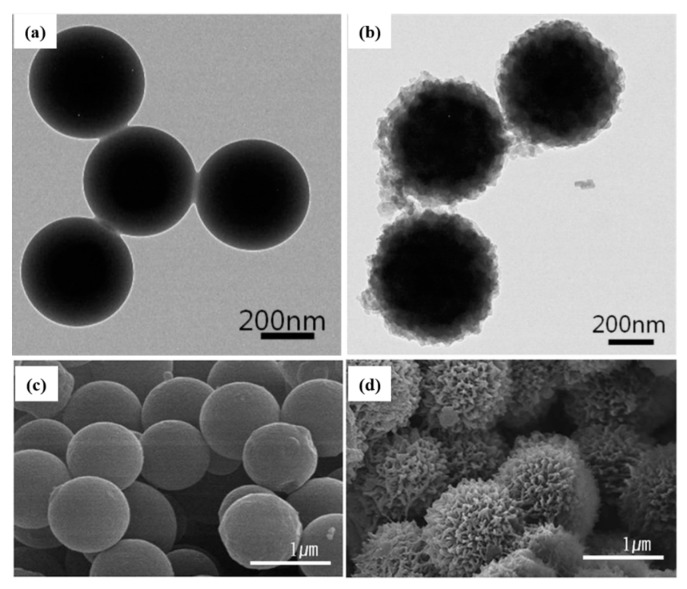
Morphologies of PANI coated polymeric nanoparticles: (**a**,**b**) TEM images of PS and PS/PANI (Piao et al. [61], © Elsevier, 2017) and (**c**,**d**) SEM images of PS and sea urchin-like PS/PANI (Kim et al. [66], © The Royal Society of Chemistry, 2015).

Piao et al. [61] synthesized core-shell PANI-coated polystyrene (PS) nanoparticles using electrostatic interactions by modifying the surface of PS using a sulfuric acid in advance. Scheme 2 shows the synthetic route of the PS/PANI nanoparticles. They claimed that the synthesized PS/PANI core-shell nanoparticles were coated better than those using a simple π-π stacking interaction [65]. Figure 1a,b shows the transmission electron microscope (TEM) photos of PS and PS/PANI, respectively. The PS exhibited a smooth boundary with a diameter of 360 nm, whereas PS/PANI exhibited a rough border with a diameter of 500 nm, which means that the PANI shell thickness is about 70 nm. The electrical conductivity of PS/PANI was 10^−3^ S/cm, but it was decreased to 10^−8^ S/cm by de-doping with a NaOH solution to make it suitable for ER materials. Kim et al. [66] synthesized sea urchin-like PS/PANI particles using a seeded swelling polymerization method, where the aniline was absorbed onto the surface of PS by π-π stacking interactions and the polymerization of aniline was performed by ferric nitrate nonahydrate to produce an urchin-like PANI shell. The urchin-like PS/PANI particles exhibited better dispersion stability than particles with a smooth surface and enhanced rheological property with their enlarged surface area than that of particles with a smooth morphology. It also demonstrated larger ER performance and increased yield stress under the same experimental conditions. Figure 1c,d present scanning electron microscope (SEM) photos of PS and urchin-like PS/PANI, respectively, where the PS had a smooth surface with a diameter of about 1 μm and the PS/PANI exhibited an urchin-like structure with a mean diameter of 1.3 μm. The conductivity of the urchin-like PS/PANI was decreased to 2.34 × 10^−11^ S/cm from 1.13 × 10^−2^ S/cm by de-doping with a NaOH solution. In addition to the nano-sized core, many PANI-functionalized micron-sized polymeric particles have been reported as ER particles. Lee et al. [67] synthesized well-controlled PANI-coated PMMA microparticles by graft polymerization, whereas Liu et al. [59] synthesized PS/PANI using a controlled releasing interfacial polymerization method. Furthermore, for comparison with isotropic spherical microspheres, Liu et al. [68] fabricated anisotropic snowman-like PMMA/PANI microparticles using a seed emulsion polymerization method, and the ER characetristics of PMMA/PANI-based ER suspensions with different particle concentrations were investigated.

##### Inorganic Core

**Figure 2:**
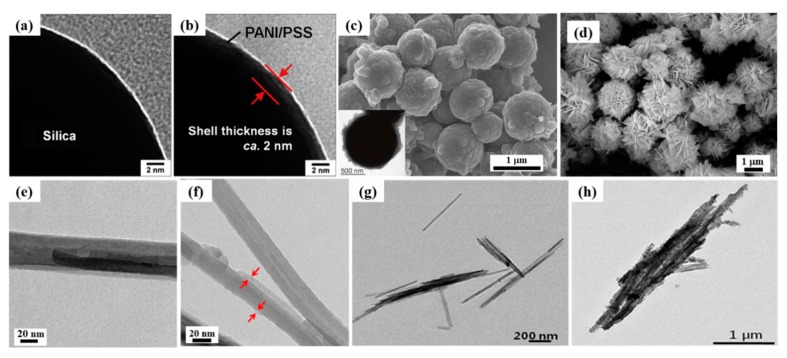
Morphologies of PANI coated inorganic nanoparticles: (**a**,**b**) SiO_2_ and PANI/PSS-coated SiO_2_ (Range between red arrows shows the thickness of PANI/PSS) (Lee et al. [70], © Elsevier, 2013); (**c**) Fe_3_O_4_/PANI (Sim et al. [72], © BME-PT, 2015); (**d**) flower-like Fe_2_O_3_/PANI (Tian et al. [73], © Elsevier, 2016); (**e**,**f**) SPL and PANI/SPL (Jang et al. [74], © Elsevier, 2015); (**g**,**h**) Pal and Pal/PANI (The red arrows point PANI) (Chae et al. [76], © Elsevier, 2007).

In the case of inorganic particles being adopted as a core part, Park et al. [69] synthesized core-shell typed SiO_2_/PANI particles using π-π stacking interactions by initially modifying the silica surface with N-[(3-trimethoxysilyl)-propyl] aniline. The diameter of SiO_2_ was approximately 1 μm and the thickness of the PANI shell in the SiO_2_/PANI was about 50 nm. The electrical conductivity of the shell was adjusted from 1.8 × 10^−2^ S/cm to 5.8 × 10^−12^ S/cm using a NaOH solution. Lee et al. [70] coated PANI/poly(styrene sulfonate) (PANI/PSS) on SiO_2_ nanoparticles with different sizes of 50, 100, and 250 nm using a seed-coating technique. The TEM images (Figure 2a,b) show that the coating layer thickness was about 2 nm. The effects of the particle size and particle concentration on the behaviors of PANI/PSS-coated SiO_2_-based ER fluid were examined. The ER property increased with increasing volume concentration and decreasing particle size. Moreover, the PANI/PSS-coated SiO_2_-based ER suspensions showed excellent dispersion stability, which also increased with decreasing article size. Wang et al. [71] synthesized TiO_2_/PANI nanoparticles using different kinds of surfactants and Brønsted acids, and the effects of the dosage of surfactant, Brønsted acid and aniline monomer on the morphology of TiO_2_/PANI particles were also investigated. Well-coated core-shell-structured TiO_2_/PANI were obtained when cetyltrimethyl ammonium bromide (CTAB) and HCl are used as a surfactant and Brønsted acid, respectively. Before being adopted as ER materials, the electrical conductivity of the synthesized TiO_2_/PANI decreased to 6.69 × 10^−9^ S/cm by a de-doping process. Sim et al. [72] coated PANI on Fe_3_O_4_ nanoparticles using electrostatic interactions and hydrogen-bond interactions by initially acidizing the Fe_3_O_4_ with an HCl solution. They reported that after the PANI coating, the surface of Fe_3_O_4_ changed significantly, and the PANI shell thickness was about 100 nm (Figure 2c). The electrical conductivity was also decreased to 5.56 × 10^−8^ S/cm by a de-doping process using a NaOH solution so it was adopted as an ER particle. Furthermore, the Fe_3_O_4_/PANI exhibited soft-magnetic properties with a saturation magnetization of 38 emu/g at a magnetic field strength of 10 kOe. Therefore, it also was assessed as an MR material. Tian et al. [73] fabricated core-shell structured flower-like Fe_2_O_3_/PANI nanoparticles by in situ polymerization using CTAB as a surfactant. SEM confirmed the flower-like morphology of the synthesized Fe_2_O_3_/PANI (Figure 2d), in which PANI was filled between the sheets and coated the sheets of the flower-like structure. The flower-like Fe_2_O_3_/PANI was also de-doped in a NH_3_·H_2_O solution to decrease the electrical conductivity.

**Scheme 3:**
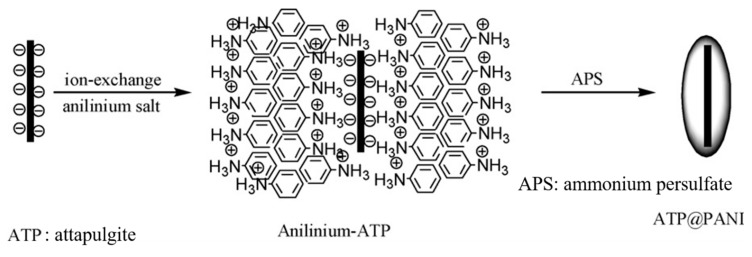
The synthesis mechanism of the PANI coated clay, (take ATP as an example) ([75], © Elsevier, 2007).

Nanofibrous clay materials, such as sepiolite (SPL), palygorskite (Pal), and attapulgite (ATP), were also used as the core of ER materials with a PANI coating. Jang et al. [74] synthesized a PANI-wrapped sepiolite composite (PANI/SPL) by in situ polymerization without a surfactant and adopted it as an ER material. The mechanism for the fabrication of PANI-coated clay was considered to be the electrostatic interactions between the clay and anilinium salt, as shown in Scheme 3. In the presence of an acid, the aniline transforms into an anilinium salt state, and the clay shows a negative center; thus, anilinium cations are adsorbed on the clay through electrostatic interactions. Furthermore, the adsorbed anilinium causes the monomer to aggregate on the clay surface due to π-π stacking interactions, and polymerize to form a PANI coating as the oxidizing agent is introduced [75]. The SPL shows a nano-needle structure with a length of 1–2.5 μm and diameter of 20–25 nm (Figure 2e). The TEM image of PANI/SPL (Figure 2f), revealed some bumps on the surface of sepiolite, which are caused by the PANI coating. Chae et al. [76] synthesized PANI-coated palygorskite (Pal/PANI) by a similar in situ polymerization process to that used for the synthesis of PANI/SPL. The Pal showed a nano-fibrous morphology with lengths ranging from the sub-micrometer to micrometer and a mean diameter of approximately 20 nm. The successful coating of PANI was confirmed in the TEM images (Figure 2g,h). The PANI-coated SPL and Pal also experienced de-doping in a NaOH solution.

#### 2.1.2. Polyaniline Derivatives

**Scheme 4:**
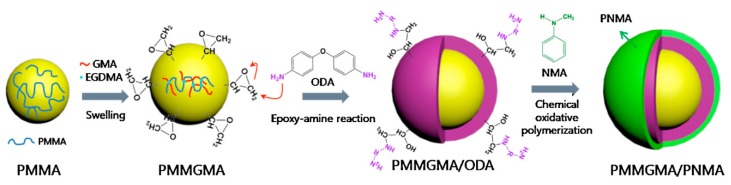
Schematic diagram of the PNMA coating on PMMA (Moon el al. [77], © Elsevier, 2015).

**Figure 3:**
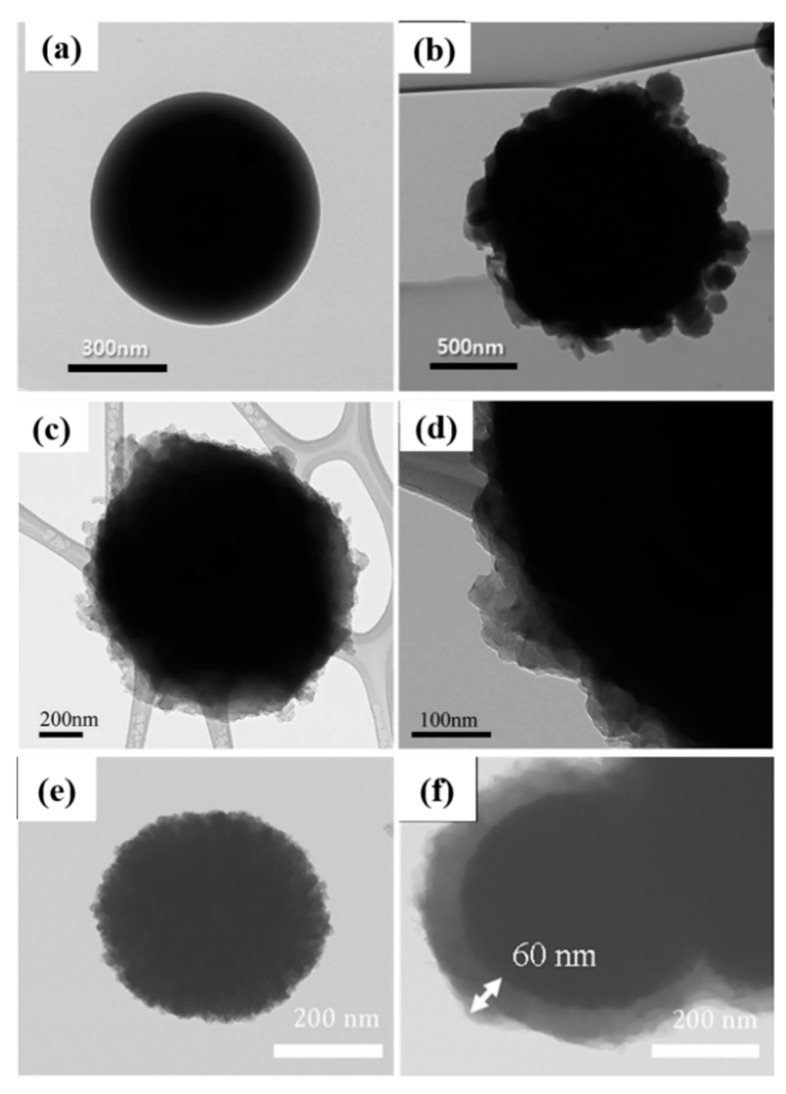
TEM images of PANI derivatives coated nanoparticles: (**a**,**b**) PMMA and PMMA-PNMA (Moon et al. [77], © Elsevier, 2015); (**c**,**d**) PEAN-PMMA (Kim et al. [78], © The Royal Society of Chemistry, 2014); (**e**,**f**) Fe_3_O_4_ and Fe_3_O_4_/POA (Lee at el. [81], © by authors, 2019).

PANI derivatives, such as polymethylanilne (PMAN) and polyethylaniline (PEAN), were further adopted as a coating shell material prepared by similar chemical oxidation polymerization. Compared to PANI, these derivatives intrinsically have several orders of magnitude lower conductivity. Therefore, composites made from PANI derivatives usually have lower electrical conductivities and could be adopted as an ER particle without undergoing a de-doping step, which is, in general, needed for PANI-based ER fluids and will certainly improve the manufacturing effectiveness. Moon et al. [77] fabricated poly(N-methylaniline) (PNMA)-coated poly(methyl methacrylate) (PMMA) by a grafting polymerization method. Scheme 4 shows the synthetic route. PMMA was first swollen by glycidyl methacrylate (GMA) then cross-linked using ethylene glycol dimethacrylate (EGDMA). The aniline group was then grafted on it by an epoxy-amine reaction between GMA and oxydianiline; finally, the N-methylaniline was adsorbed by π-π stacking interactions and formed PNMA by oxidative chemical polymerization. The PMMA seed showed a diameter of 700 nm. After swelling and coating, the diameter of PMMA-PNMA was approximately 1.63 μm and the thickness of PNMA was approximately 100 nm (Figure 3a,b). The conductivity of the as-fabricated PMMA-PNMA was 9.319 × 10^−4^ S/cm, which was relatively lower than that of PANI-coated materials (usually higher than 10^−3^ S/cm), but this value is still high for the application of ER materials. Consequently, it was also adjusted to 5.35 × 10^−10^ S/cm by de-doping. Kim et al. [78] fabricated poly(2-ethylaniline) (PEAN)-coated PMMA by changing the 2-ethylaniline (NMA) monomer to an EAN monomer in the same synthetic route as PMMA-PNMA. The particle size and conducting polymer thickness of PEAN-PMMA (Figure 3c,d) were similar to those of PMMA-PNMA. The most important point is that PEAN-PMMA was applied directly to the ER material without undergoing any de-doping process and showed excellent ER performance. Furthermore, Kwon et al. [79] fabricated poly(2-methylaniline)-coated PS (PS/PMAN) particles as ER materials by employing a controlled swelling-releasing method to PS seeds with a diameter of 1.3 μm. The thickness of PMAN was about 50 nm, and their electrical conductivity was 10^−10^ S/cm, which is an appropriate value for an ER material. Lee et al. [80] fabricated poly(o-anisidine)-coated silica (SiO_2_/POA) by modifying the surface of SiO_2_ using N-[(3-trimethoxysilyl)-propyl] aniline in a similar manner to SiO_2_/PANI synthesized by Park et al. [69]. Both SiO_2_ and SiO_2_/POA have a mean diameter of 1 μm and the POA layer thickness in SiO_2_/POA is approximately 50 nm. The electrical conductivity of SiO_2_/POA was 3.6 × 10^−7^ S/cm, which is quite low compared to PANI-coated particles, and such a value makes it possible for the SiO_2_/POA to be used directly in the ER fluid without de-doping. In addition, POA-coated Fe_3_O_4_ was also fabricated and adopted as an MR material [75]. The thickness of the coated POA was approximately 60 nm (Figure 3e,f), and the density of Fe_3_O_4_/POA was 2.52 g/cm^3^, which was lower than that of Fe_3_O_4_ (4.34 g/cm^3^). Therefore, the Fe_3_O_4_/POA-based ER fluid showed better dispersion stability than Fe_3_O_4_, accompanied by excellent MR performance.

### 2.2. Other Conducting Polymers

Although PANI and its derivatives have been adopted as the conducting shell parts of core-shell structured ER particles, other conducting polymers, such as PPy, polythiophene (PT), and PPP have also been considered to be potential ER materials because of its excellent conductivity and exceptional environment stability [82,83,84,85]. In the case of these conducting polymer-coated nanoparticles, PEDOT as a derivative of PT, is one of the most successfully adopted conducting polymers owing to their air stability, low band gap, optical transparency, and high conductivity [86].

**Figure 4:**
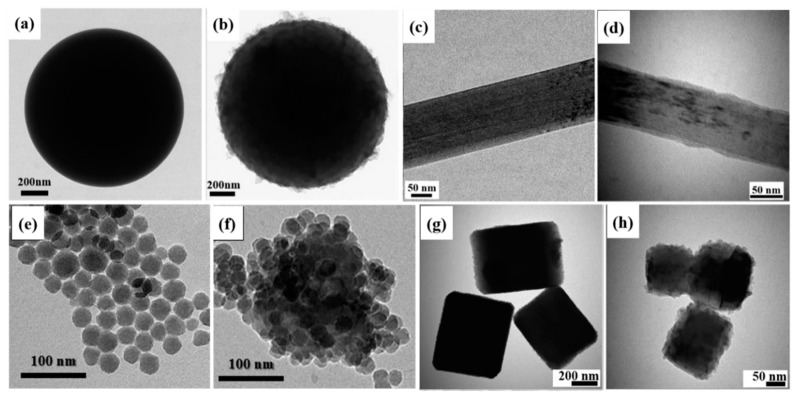
TEM images of (**a**,**b**) PS and PS/PEDOT (An et al. [87], © Elsevier, 2007); (**c**,**d**) nanorod-TiO_2_ and nanorod-TiO_2_/PEDOT (Eroal et al. [88], © The Royal Society of Chemistry, 2015); (**e**,**f**) SiO_2_ and PEDOT/SiO_2_ (Park et al. [89], © Elsevier, 2018); (**g**,**h**) nanocube-TiO_2_ and nanocube-TiO_2_/P3OT (Sever et al. [90], © Elsevier, 2015).

**Scheme 5:**
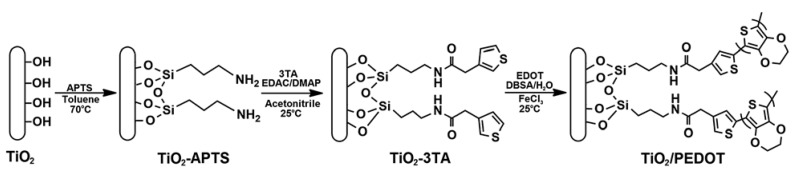
Schematic diagram of the synthetic route of TiO_2_/PEDOT (Eroal et al. [88], © The Royal Society of Chemistry, 2015).

An et al. [87] synthesized PS/PEDOT microspheres as an ER material via a swelling-diffusion interfacial polymerization technique, providing a uniformly coated morphology and structure. The PS/PEDOT shows a mean diameter of approximately 1 μm with a PEDOT thickness of approximately 10 nm (Figure 4a,b). The electrical conductivity after de-doping decreased from 10^−4^ S/cm to 10^−8^ S/cm before being used as an ER particle. Eroal et al. [88] fabricated core-shell shaped TiO_2_/PEDOT by covalent bonding using TiO_2_ nanorods and nanoparticles as the core, respectively. As shown in Scheme 5, initially, amino-functionalized TiO_2_ was obtained by the surface silanization of TiO_2_ using 3-aminopropyltriethoxysilane (APTS), and the thiophene group was then grafted using 3-thiophene acetic acid to carry out polymerization with EDOT. The covalently bonded TiO_2_/PEDOT is believed to contribute a stronger interaction between the two components. Figure 4c,d shows TEM images of nanorod-TiO_2_ and nanorod-TiO_2_/PEDOT, respectively. Nanorod-TiO_2_ showed a smooth surface with lengths on the micrometer scale and diameters of approximately 30–100 nm, while nanorod-TiO_2_/PEDOT exhibited a distinct convex shell with a thickness of 6–7 nm. Prior to adoption as an ER particle, the conductivity of the nanoparticle-TiO_2_/PEDOT and nanorod-TiO_2_/PEDOT decreased from 2.63 and 2.69 S/cm to 6.8 × 10^−2^ and 7.8 × 10^−2^ S/cm, respectively. The nanorod-TiO_2_/PEDOT-based ER fluid showed better ER efficiency than the nanoparticle-TiO_2_/PEDOT-based ER suspension. Furthermore, the nanorod-TiO_2_/PEDOT-based ER suspension with the 5 vol % concentration showed excellent dispersion stability with no sedimentation. Park et al. [89] synthesized PEDOT-wrapped SiO_2_ by in situ polymerization without any surfactant and applied it as an ER material. SiO_2_ (Figure 4e) exhibited a spherical morphology with a diameter ranging from 10 to 50 nm, while after the PEDOT coating, the PEDOT/SiO_2_ (Figure 4f) showed a PEDOT-wrapped cluster of SiO_2_ with a mean diameter of 300 nm. The electrical conductivity of PEDOT/SiO_2_ was 10^−6^ S/cm, which decreased to 10^−8^ S/cm for application as an ER material.

On the other hand, like other conducting polymers, PEDOT has several drawbacks, such as insolubility in typical organic solvents. This issue was improved by introducing poly(styrene sulfonic acid) (PSS) as a dopant during the polymerization of ethylenedioxythiophene so that the PEDOT/PSS dispersion can be stable with its high electrical conductivity [91]. Liu et al. [92] synthesized PEDOT/PSS/PS microspheres using a simple physical adsorption method and applied them as an ER particle. Kim et al. [93] applied PEDOT:PSS as a solid surfactant for Pickering emulsion polymerization to synthesize PS/PEDOT:PSS composite particles with an average diameter of 14.2 μm. Note that the Pickering emulsion process is superior to typical emulsion techniques because solid particle stabilizers are replaced for organic surfactants to prevent agglomeration during the emulsion process.

As another derivative of PT, poly(3-octylthiophene) (P3OT), was also used to coat nanocube-TiO_2_ for use as an ER material [90,94]. The P3OT coating was bonded covalently, which is similar to the route of TiO_2_/PEDOT synthesized by Eroal et al. [88]. Figure 4g,h shows TEM images of nanocube-TiO_2_ and nanocube-TiO_2_/P3OT, respectively. The nanocube-TiO_2_ exhibited a cubic morphology with a particle size ranging from 300 to 700 nm, and the thickness of the P3OT layer of nanocube-TiO_2_/P3OT was approximately 12–30 nm. The electrical conductivity of nanocube-TiO_2_ and nanocube-TiO_2_/P3OT was 0.22 × 10^−5^ S/cm and 1.16 × 10^−5^ S/cm, respectively. The ER characteristics of the dispersions consisted of nanocube-TiO_2_ and nanocube-TiO_2_/P3OT suspended in silicone oil with a particle volume fraction of 1.25–7.5% were investigated. The nanocube-TiO_2_/P3OT-based ER suspension demonstrated better ER performance than nanocube-TiO_2_. Moreover, the dispersion stability of the nanocube-TiO_2_/P3OT-based ER suspension was superior to that of the nanocube-TiO_2_ based ER fluid; the anti-sedimentation ratios of them were 65% and 31%, respectively.

**Figure 5:**
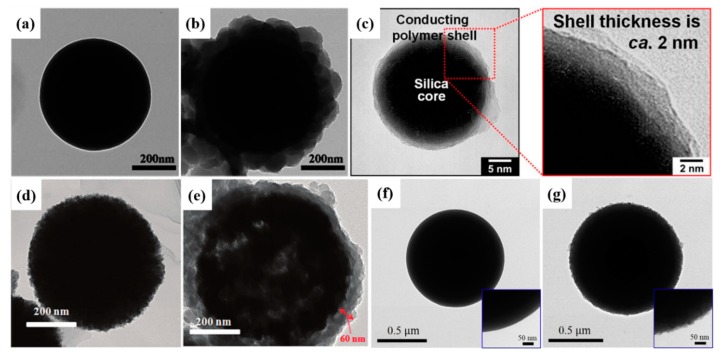
TEM images of (**a**,**b**) SiO_2_ and SiO_2_/PPy synthesized by Kim et al. [95] (© The Royal Society of Chemistry, 2016); (**c**) SiO_2_/PPy synthesized by Hong et al. [96] (© The Royal Society of Chemistry, 2010); (**d**,**e**) Fe_3_O_4_ and Fe_3_O_4_/PPy (Park et al. [97], © The Royal Society of Chemistry, 2015); (**f**,**g**) PS and PS/PDPA (Kim et al. [98], © Elsevier, 2017).

**Scheme 6:**
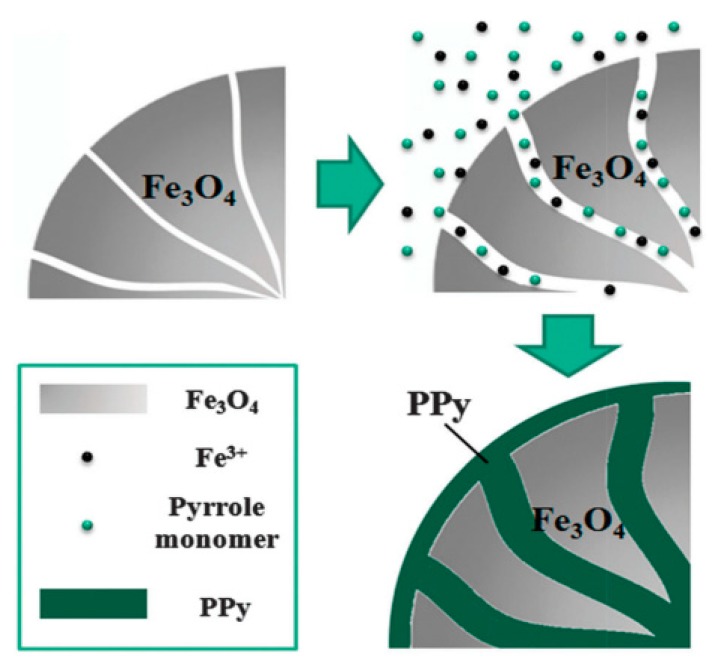
Polymerization process of Fe_3_O_4_ composite microspheres (Park et al. [97], © Journal of Materials Chemistry C, 2015).

Furthermore, Kim et al. [95] synthesized PPy-coated SiO_2_ (SiO_2_/PPy) by previously modifying the surface of SiO_2_ with 3-(trime-thoxysilyl)propyl methacrylate (MPS). The SiO_2_ (Figure 5a) showed a smooth surface with a diameter of 500 nm. On the other hand, the TEM image of SiO_2_/PPy (Figure 5b) revealed a raised PPy shell with a thickness of approximately 90 nm. The electrical conductivity of the as-synthesized SiO_2_/PPy was 2.3 × 10^−8^ S/cm, which is an appropriate value for applying it as an ER material. Hong et al. [24] synthesized PT-coated SiO_2_ (SiO_2_/PT) with different particle sizes of 11, 16, and 26 nm using a seeded polymerization method by employing different sized SiO_2_ nanoseeds (7, 11, and 22 nm). The thickness of the PT layer on SiO_2_ was about 2 nm, and the electrical conductivity was 10^−7^ S/cm, which is an appropriate value as ER materials without de-doping. The ER properties of different sized-SiO_2_/PT-based ER fluids with a particle volume fraction ranging from 5 to 30 vol % were investigated. The ER efficiency increased with increasing volume fraction and decreasing particle size. The ER fluid based on 11 nm SiO_2_/PT with a concentration of 30 vol % showed a dynamic yield stress of up to 3.9 kPa at an electrical field strength of 3.0 kV/mm. Furthermore, they investigated the ER properties of the ER suspensions based on various conducting polymer-coated SiO_2_ materials [96], including PT, PPy, PEDOT, and PANI. The size of the SiO_2_ material was 22 nm and the thickness of the conducting polymer layer was 2 nm. Figure 5c presents TEM images of SiO_2_/PPy. The ER efficiency of these particle-based ER fluids showed an order of SiO_2_/PANI > SiO_2_/PEDOT > SiO_2_/PPy > SiO_2_/PT. The change in ER efficiency might related to the charge transport behavior of the conducting polymer shell. In other words, the optical band gap energy and charge carrier mobility of the conducting polymer were considered to be key parameters for determining the ER efficiency. In addition, Park et al. [97] synthesized Fe_3_O_4_-PPy composites through in situ polymerization and adopted it as ER and MR particles. Fe_3_O_4_ was initially prepared by FeCl_3_·6H_2_O, sodium acetate, and ethylene glycol. In the polymerization process, the Fe^3+^ ion is separated from the surface of Fe_3_O_4_, and the pyrrole monomers act as an initiator for polymerization at the exterior shell of the Fe_3_O_4_ particles, which is well described in Scheme 6. The PPy coating was observed by TEM imaging (Figure 5d,e) of the composite. The black part of Fe_3_O_4_ was coated with PPy, which is shown as an internal black core in the TEM image. Accordingly, the hollow portion of the Fe_3_O_4_-PPy was observed, which makes it a special rather than simple core-shell structure. The mean diameter of Fe_3_O_4_ and Fe_3_O_4_-PPy from dynamic light scattering (DLS) measurements was 456.6 nm and 578.8 nm, respectively. The thickness of the PPy shell was 60 nm, which is similar to the thickness calculated from the TEM image. For use in an ER fluid, the conductivity of Fe_3_O_4_/PPy was adjusted to 10^−7^ S/cm from 10^−2^ S/cm. The Fe_3_O_4_/PPy showed soft-magnetic properties with a saturation magnetization of 41 emu/g. Therefore, the Fe_3_O_4_/PPy was also adopted in the MR fluid, and the MR properties were investigated.

As another potential conducting polymer, poly(diphenylamine) (PDPA) has recently been employed as potential ER material owing to its excellent optical properties, environmental stability, ease of synthesis, and suitable electrical conductivity [99,100]. Kim et al. [98] introduced PDPA as a conducting polymer shell onto PS by π-π stacking interactions to fabricate a core-shell structured particle and adopted it as an ER material without de-doping. The particle size of PS and PS/PDPA was approximately 1 μm (Figure 5f,g), and the thickness of the thin PDPA layer calculated from thermogravimetric analysis was approximately 16.12 nm.

## 3. Thermal Properties

**Figure 6:**
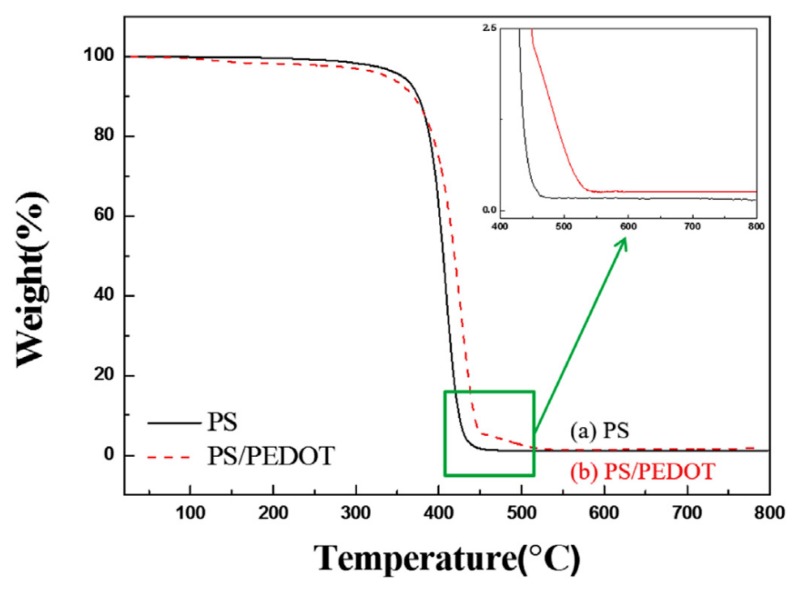
TGA curves of (**a**) PS and (**b**) PS/PEDOT (An et al. [87], © Elsevier, 2007).

Thermogravimetric (TGA) analysis is usually performed because the thermal stability of a material has an important influence on its practical applications in industry; Figure 6 presents the TGA curves of PS/PEDOT from 25 to 800 °C at a heating rate of 10 °C/min under a N_2_ atmosphere. The PS began to show a one-step decrease in mass at approximately 360 °C caused by thermal degradation of the PS chain backbone. Meanwhile, because of the PEDOT coating, the PS/PEDOT showed a two-step decrease at approximately 320 and 450 °C, respectively. On the other hand, based on the thermal degradation behavior studied, the coated conducting layer thickness can be estimated from the mass loss of each component through the followed mass balance equation [101]:

(1a) $$\frac{V_{2}\rho_{2}}{V_{1}\rho_{1}{\;+\;V}_{2}\rho_{2}}\;\times \;100\%\;=\;m$$

(1b) $$V_{1}=\frac{4}{3}{\pi R}^{3}$$

(1c) $$V_{2}=\frac{4}{3}\pi \left({\left(R\;+\;d\right)}^{3}-R^{3}\right)$$

Here, V and ρ represent the volume and density of the core (index 1) and shell (index 2), respectively. m is the mass ratio of the shell, which can usually be obtained from the TGA curves. *R* is the core radius, and d indicates the shell thickness. Using PS/PEDOT as an example, the diameter of PS was 1.05 μm, i.e., R ≈ 525 nm. The density of PS and PEDOT were determined to be 1.083 ($\rho_{1}$) and 1.65 g/cm^3^ ($\rho_{2}$), respectively. From the TGA curve, the mass ratio of PEDOT can be analyzed to be approximately 5.519% (m). Therefore, *d* can be calculated as approximately 6.63 nm. Note that this approach can be applied to the case of monodisperse spherical core particles with a uniform coating layer; otherwise, an obvious error can occur. In most cases, however, the thickness of the coated shell estimated from this indirect method showed good agreement with that observed in the direct TEM images [65,102].

## 4. Sedimentation Stability

**Figure 7:**
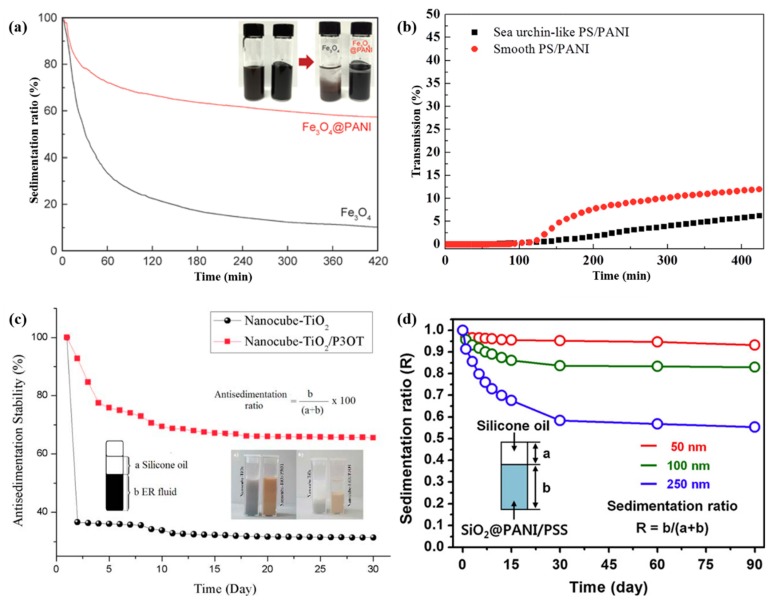
Sedimentation stabilities of suspensions composed by (**a**) Fe_3_O_4_ and Fe_3_O_4_/PANI (Sim et al. [72], © BME-PT, 2015); (**b**) smooth PS/PANI and sea urchin-like PS/PANI (Kim et al. [66], © The Royal Society of Chemistry, 2015); (**c**) nanocube-TiO_2_ and nanocube-TiO_2_/P3OT (Sever et al. [94], © Society of Plastics Engineers, 2016); (**d**) PANI/PSS-coated SiO_2_ with different size (Lee et al. [70], © Elsevier, 2013).

From a potential engineering application point of view, the sedimentation stability of an ER and MR fluid is very important, and is related to various factors, including the density mismatch, compatibility, and interactions between the particles and carrier liquid, as well as the shape, size, and surface characteristics of the particles [103,104]. In general, especially for some high-density inorganic materials, the density of a composite can be reduced considerably when coated with a conductive polymer, which generally favors better dispersion stability of the material in the dispersed phase. Figure 7a shows the sedimentation ratio of Fe_3_O_4_ (ρ = 4.46 g/cm^3^) and Fe_3_O_4_/PANI (ρ = 2.76 g/cm^3^) suspended in silicone oil with a particle concentration of 20 wt %, in which the sedimentation ratio was calculated from the transmission data examined using a Turbiscan. Fe_3_O_4_/PANI exhibits superior sedimentation stability to Fe_3_O_4_, which is due mainly to the smaller density mismatch between Fe_3_O_4_/PANI and oil. The same conclusion was also verified in a study of Fe_3_O_4_/POA-based MR fluids [81]. The transmission of ER fluid based on PS/PANI with a smooth surface and a sea urchin-like structure was investigated as a function of time using a Turbiscan. Here, higher transmission indicates more severe particle sedimentation. Moreover, the sea urchin-like PS/PANI with a rough surface showed better dispersion stability than the PS/PANI with a smooth surface, as shown in Figure 7b. Furthermore, the particle sizes and densities of the two types of PS/PANI particles were similar. Therefore, the better sedimentation stability of the sea urchin-like PS/PANI can be attributed to the larger drag force and inter-particles frictional force from the high specific surface area of urchin-like shape. Figure 7c shows the anti-sedimentation ratio of nanocube-TiO_2_ and nanocube-TiO_2_/P3OT-based ER fluids as a function of time, where the anti-sedimentation ratio was defined as:

(2) $$\mathrm{Anti}-\mathrm{sedimentation}\;\mathrm{ratio}\;=\;\;\frac{b}{\left(a\;+\;b\right)}\;\times \;100\%$$

where a and b represent the thicknesses of the supernatant and the precipitate layer, respectively. This means that the sedimentation stability of the suspension improves with increasing anti-sedimentation ratio. As shown, the anti-sedimentation ratios of the nanocube-TiO_2_ and nanocube-TiO_2_/P3OT after 30 days were 31% and 65%, respectively. In addition, the anti-sedimentation ratios of nanosphere-TiO_2_ and nanosphere-TiO_2_/PPy were 15% and 54%, respectively [105]. Furthermore, the densities of the nanocube-TiO_2_, nanocube-TiO_2_/P3OT, nanosphere-TiO_2_, and nanosphere-TiO_2_/PPy were 3.4, 3.37, 3.88, and 2.8 g/cm^3^, respectively. The density of the nanocube-TiO_2_/P3OT was higher than the nanosphere-TiO_2_/PPy, while the former showed better sedimentation stability. Therefore, it was proposed that particles with a cubic morphology could contribute better sedimentation stability than spheres. In addition, in the study of nanorod-TiO_2_/PEDOT and nanoparticle-TiO_2_/PEDOT-based ER fluid [88], it was also found that the TiO_2_/PEDOT with a rod shape has better sedimentation stability than that with a spherical shape. Moreover, the sedimentation stability increased with increasing particle volume fraction. Rod-like particles have a high specific surface area to enhance the interaction among particles, and that the entanglement structure of the particles can be formed because the rotational motion of the rod-like particles is limited, thereby reducing the sedimentation speed of the particles in the liquid. In addition, an increase in particle concentration causes an increase in particle-particle interaction and particle-liquid interaction, thereby improving the stability of the sedimentation. Furthermore, if the mutual interference between the particles is neglected, in the case of spherical particles with a smooth surface that are dispersed uniformly in a viscous fluid and with a small Reynolds number (laminar flow), the sedimentation velocity of the particles can be expressed by Stoke’s law [106] as follows:

(3) $$v\;=\;\frac{{2R}^{2}\left(\rho_{p}-\rho_{f}\right)g}{9\eta }$$

where v represents the sedimentation velocity; R is the radius of particles; $\rho_{p}$ and $\rho_{f}$ are the densities of the particle and medium oil, respectively; g is the gravitational acceleration, and η is the dynamic viscosity of a fluid. According to Equation (3), the sedimentation velocity is closely related to the deference in density between the particles and the liquid, as well as the particle size. That is, the small density difference and particle diameter can contribute to the relatively high sedimentation stability. Figure 7d shows the sedimentation properties of PANI/PSS-coated SiO_2_ spheres suspended in silicone oil with different sizes of 50 nm (ρ = 2.57 g/cm^3^), 100 nm (ρ = 2.62 g/cm^3^), and 250 nm (ρ = 2.64 g/cm^3^), respectively. As shown, the PANI/PSS-coated SiO_2_ spheres with a diameter of 50 nm, and a density of 2.57 g/cm^3^ exhibited the best sedimentation stability, which is consistent with the prediction.

## 5. Dielectric Properties

Dielectric property of ER materials is important, particularly considering the polarization mechanism of the ER fluids. Their dielectric spectrum is generally obtained experimentally using an LCR meter to understand their ER output further. The Cole–Cole equation given in the Equation (4), which is useful for revealing the relationship between the dielectric characteristics and the ER performance, was adopted to fit the dielectric data and explain the dielectric characteristics. The Cole-Cole model is presented as follows:

(4) $$\varepsilon^{*}{=\varepsilon }^{\prime }{–\;i\varepsilon }^{\prime \prime }{=\varepsilon }_{\infty }-\frac{\varepsilon_{0}{\;–\;\varepsilon }_{\infty }}{1\;+\;{\left(i\omega \lambda \right)}^{1\;–\;\alpha }}\;\left(0\;\leq \;\alpha \;<\;1\right)$$

where $\varepsilon^{*}$ is the complex permittivity; $\varepsilon^{\prime }$ and $\varepsilon^{\prime \prime }$ are the dielectric constant and dielectric loss factor, respectively; $\varepsilon_{0}$ and $\varepsilon_{\infty }$ are the static and infinite frequency dielectric constants; ω is the angular frequency; λ is the relaxation time of interfacial polarization, which is denoted by $\lambda \;=\;1/{2f}_{max}$ and $f_{max}$ is the frequency at which the $\varepsilon^{\prime \prime }$ shows the maximum value. The exponent, 1 – α, indicates the broadness of the relaxation time distribution.

**Figure 8:**
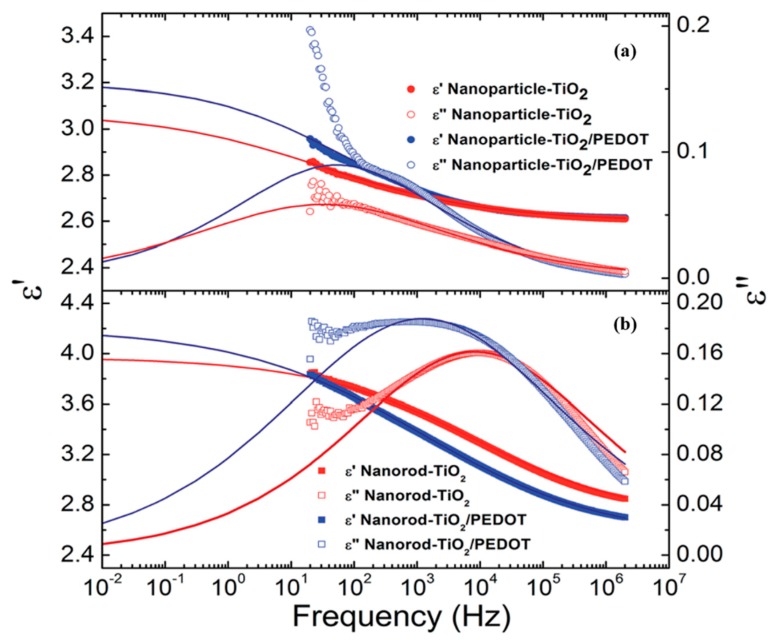
Dielectric spectra of (**a**) nanoparticle-TiO_2_ and nanoparticle-TiO_2_/PEDOT; (**b**) nanorod-TiO_2_ and nanorod-TiO_2_/PEDOT (Eroal et al. [88], © The Royal Society of Chemistry, 2015).

Figure 8 shows the dielectric spectra of the ER fluids based on nanoparticle-TiO_2_, nanoparticle-TiO_2_/PEDOT, nanorod-TiO_2_, and nanorod-TiO_2_/PEDOT, respectively, and the fitted lines are generated from Equation (4). Table 1 shows the fitted parameters, in which $\Delta {\varepsilon \;=\;\varepsilon }_{0}{\;–\;\varepsilon }_{\infty }$ indicates the achievable polarization degree, and λ relates to the polarization rate. The values of Δε and λ play an important role in predicting the ER performance of ER fluids. In general, a high Δε value and a short λ are believed to contribute to the building-up of a more stable chain form under an electrical field to show high ER performance. As shown in Table 1, the nanorod-TiO_2_ and nanorod-TiO_2_/PEDOT showed higher Δε and shorter λ values than nanoparticle-TiO_2_ and nanoparticle-TiO_2_/PEDOT, and as expected, the nanorod-structured particles exhibited higher ER performance. Furthermore, a comparison of nanorod-TiO_2_ and nanorod-TiO_2_/PEDOT showed that the Δε value of nanorod-TiO_2_/PEDOT is higher than that of nanorod-TiO_2,_ but λ is lower. In contrast, the ER performance of nanorod-TiO_2_/PEDOT is higher than nanorod-TiO_2_, which suggests that high Δε makes a greater contribution to a high active ER fluid than λ. On the other hand, when the value of Δε is high, the value of λ is low, which is not always associated with higher ER effects. Here, nanoparticle-TiO_2_/PEDOT has a higher Δε value and shorter λ than nanoparticle-TiO_2_, which exhibits a lower ER effect. Therefore, the dielectric properties are important for ER effects, but other factors should also be considered when analyzing the ER effects, such as the particle morphology, size, and surface characteristics.

## 6. Electrorheological Characteristics

### 6.1. Electrorheological Phenomenon

Winslow first discovered that the ER behavior was caused by the chain formation of wet particles with a proper net charge in an insulating medium oil [107], and proposed the so-called “water bridge model”. Nevertheless, several mechanisms for wet-based hydrous ER materials could not be expanded to dry-base materials. For anhydrous ER suspensions, a polarization model was introduced to explain the chain formation in the presence of an electrical field that originated from dielectric mismatch between the dispersed electro-responsive particles and carrier oil [108]. This model is good at a relatively low applied electrical field strength and low particle concentration. For high particle concentrations in ER fluids, however, the distance between the conductive particles in the medium oil decreases and the electrical output of the ER suspension becomes nonlinear, often demonstrating electric breakdown or particle discharge at a high electrical field. In this case, the ER phenomenon is produced by the medium-induced conductivity increment between most touching particles. The electrical conductivity difference between the particle and medium oil, rather than dielectric constant difference, is considered a major factor for the fibrillation of dispersed particles under an input electric field. This conduction model considers only the particle interaction, regardless of the microstructural change after an electrical field is induced [109]. In contrast, while the polarization model predicts a slope of 2.0 for the yield stress as a function of the external electrical field strength, the conduction model provides a slope of 1.5. For many ER fluids, however, the slope often deviates from these values so that a hybrid equation is needed to cover the yield stress over a wide range of electrical field strength and particle concentrations, as will be discussed in Section 6.2 [110].

**Figure 9:**
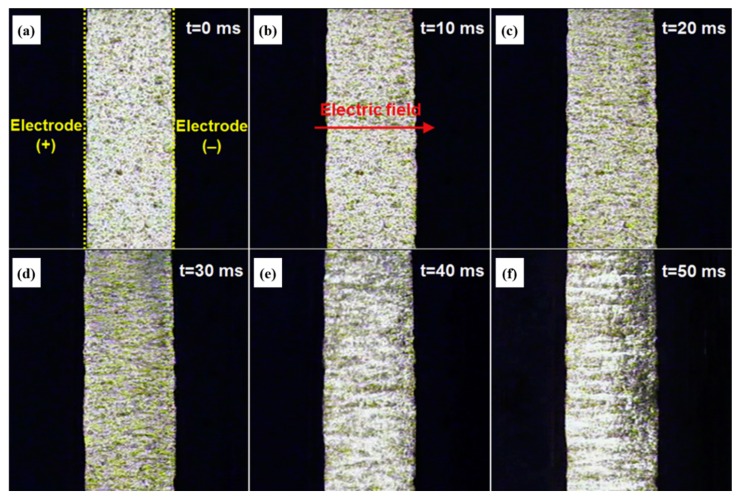
Optical microscope images of PANI/PSS-coated silica spheres dispersed in silicone oil (30 vol %) under applied electric field with different response time of (**a**) 0, (**b**) 10, (**c**) 20, (**d**) 30, (**e**) 40 and (**f**) 50 ms. The gap distance between the electrodes was adjusted to 1 mm (Lee et al. [70], © Elsevier, 2013).

Furthermore, to confirm the ER phenomenon directly, the micro-structural changes in the conducting polymer-coated nanoparticle-based ER suspension were detected by optical microscopy (OM) under an input DC electric field, as shown in Figure 9. Without an electric field, the fabricated particles were suspended randomly in the medium oil, exhibiting a liquid-like behavior. As soon as the electrical field strength was switched on, the dispersed particles immediately began to move parallel to the direction of the input electrical field between the electrodes, taking only a few tens of milliseconds to build chain-like structures. The aligned chain-like structures facilitated swift structural reformation under a shear force in addition to increased flow resistance to shear.

### 6.2. Steady Shear Tests

**Figure 10:**
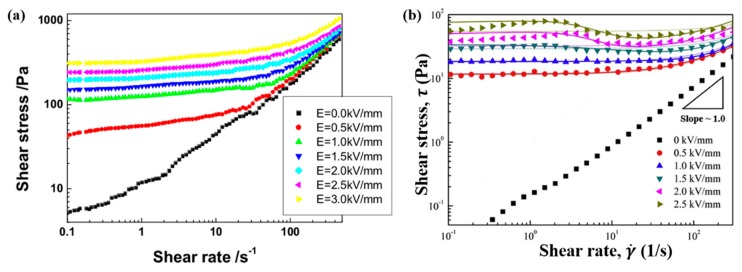
Shear stress curves of (**a**) flower-like Fe_2_O_3_/PANI based ER fluid (20 wt %) (Tian et al. [73], © Elsevier, 2016) and (**b**) PS/PDPA based ER fluid (5 vol % (Kim et al. [98], © Elsevier, 2017).

The rheological behaviors of ER fluids, which are generally measured via a steady simple shear and a dynamic mode using a rotation rheometer under an input electrical field, are quite complicated compared to typical suspension systems. The steady shear test observes the flow curves in either a controlled shear rate (CSR) or controlled shear stress (CSS) method. The flow curve of the ER fluids is determined in CSR mode, i.e., the shear stress (*τ*) and shear viscosity (*η*) as a function of the shear rate ($\dot{\gamma }$). Moreover, the dynamic yield stress is estimated by extrapolating the τ at a zero $\dot{\gamma }$ limit from the shear stress curve. Figure 10a represents the shear stress curves of flower-like Fe_2_O_3_/PANI-based ER fluid, which behaved similarly to a Newtonian fluid without an electrical field, in which the *τ* increased monotonically with increasing $\dot{\gamma }$ [111]. When the electrical field was employed, however, the yield stress appeared, and the *τ* maintained a stable plateau over a wide $\dot{\gamma }$ region, indicating Bingham fluid behavior [112]. In general, the Bingham plastic model is used widely to describe the relationship between the τ and η of the ER fluids under an electric field as follows:

(5) $$\tau =\;\tau_{y}+\eta_{pl}\dot{\gamma },\;\tau \geq \tau_{y}\;\dot{\gamma }=0,\tau <\tau_{y}$$

where $\tau_{y}$ is the dynamic yield stress, which is related to the electric field strength and $\eta_{pl}$ represents the plastic viscosity, which is generally close to the viscosity at which the $\dot{\gamma }$ tends to be infinite. In many cases, however, the shear stress curve degrades at low shear rates, and the Bingham model is unable to account for the curve. Therefore, a modified rheology equation with six parameters called the Cho–Choi–Jhon (CCJ) model, was introduced to explain this type of flow curve [11], which can be represented as follows:

(6) $$\tau =\;\frac{\tau_{y}}{1+{\left(t_{1}\dot{\gamma }\right)}^{\alpha }}+\eta_{\infty }(1+\frac{1}{{\left(t_{2}\dot{\gamma }\right)}^{\beta }})\dot{\gamma }$$

Here, $\eta_{\infty }$ is the infinite shear viscosity. $t_{1}$ and $t_{2}$ are time constants. The parameter, *α*, is related to the decrease in the *τ*. The exponent, β, has the range, 0 < β ≤ 1, because $d\tau /d\dot{\gamma }\geq 0$ is above the critical $\dot{\gamma }$ at which the τ becomes a minimum. The unusual behavior of ER fluids usually corresponds better to the CCJ equation than other models, and the decrease in τ was explained from the mechanism in a low shear rate area. Figure 10b presents the shear stress curves of the PS/PDPA-based ER fluid as a function of the $\dot{\gamma }$ under various electrical field strengths. The CCJ model explains the curves better than the Bingham equation.

**Figure 11:**
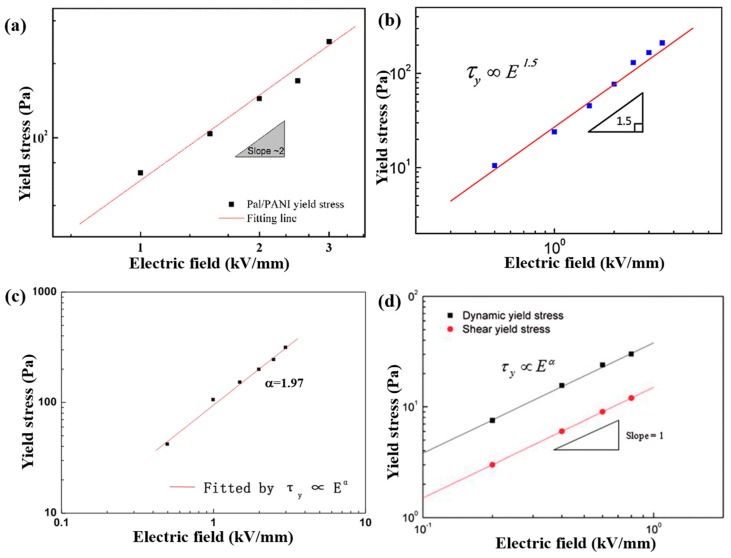
Dynamic yield stress as a function of electric field strength for ER fluids based on (**a**) Pal/PANI (10 vol %) (Chae et al. [76], © Elsevier, 2015), (**b**) PMMA-PNMA (10 vol %) (Moon et al. [77], © Elsevier, 2015), (**c**) flower-like Fe_2_O_3_/PANI (20 wt %) (Tian et al. [73], © Elsevier, 2016) and (**d**) Fe_3_O_4_/PPy (10 vol %) (Park et al. [97], © The Royal Society of Chemistry, 2015).

On the other hand, the $\tau_{y}$ is defined as the minimum stress that can continuously breakdown the chain-like structure reformed due to the external electric field, which can usually be estimated by extrapolating the shear stress to a zero limit $\dot{\gamma }$ or by fitting to models, such as Bingham and CCJ. Furthermore, the dependence of the $\tau_{y}$ on the electrical field is not only important for the engineering applications of ER fluids but also a theoretical understanding of ER fluids. Generally, the relation between the yield stress and electrical field can be noted as:

(7) $$\tau_{y}\propto E^{\alpha }$$

where E is the electrical field strength, and the index α usually ranges from 1.0 to 2.0. α = 2.0, when the ER fluids follow the polarization model, and α = 1.5, when it follows the conduction model. Figure 11a,b show the dynamic yield stress of Pal/PANI and PMMA-PNMA-based ER fluids as a function of the E, respectively. The Pal/PANI-based ER suspension showed a slope of 2.0, which proves that it conforms to the polarization model.

In contrast, the PMMA-PNMA-based ER fluid showed a slope of 1.5, which is consistent with the conduction model. Meanwhile, these theoretical models are based on the assumption that the particles dispersed in the ER fluid are monodispersed and spherical but, in fact, the yield stress of the ER suspension is actually affected by other factors, such as the shape, size, and surface characteristics of the particles and particle concentration. Therefore, in many practical cases, the ER fluid does not completely follow the polarization model or the conductive model. As given in Figure 11c,d, the slope of flower-like Fe_2_O_3_/PANI and Fe_3_O_4_/PPy-based ER fluids were 1.97 and 1.0, respectively, which neither follow the polarization nor the conduction model.

### 6.3. Dynamic Oscillation Tests

**Figure 12:**
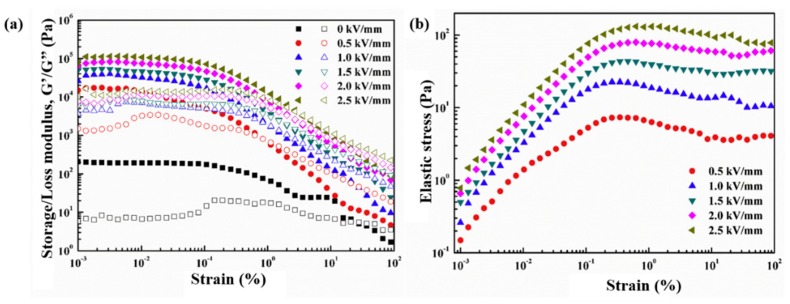
(**a**) Strain amplitude sweep test (closed symbol: *G′*, open symbol: *G″*) and (**b**) elastic stress (*τ′ = G′γ*) as a function of strain of PS/PANI based ER fluid (10 vol %) (Piao et al. [61], © Elsevier, 2017).

In addition to the steady shear measurements of ER suspensions using a rotation rheometer with an input E, the viscoelastic property is also important for their application. Generally, ER fluid is viscous without an electric field, while it transforms into a solid-like phase under an input E. To perform the dynamic test, strain amplitude sweep tests are initially performed to find a linear viscoelastic (LVE) region at a constant ω in the strain range. In Figure 12a, the storage (*G′*) and loss modulus (*G″*) are given as a function of the strain (*γ*), ranging from 0.001%–100% with a constant ω of 6.28 rad/s under electric field strengths. With an input E, *G*′ and *G″* increase by several orders of magnitude. In addition, at a low *γ* region, *G′* and *G″* show a constant plateau, which is denoted by $\gamma_{LVE}$. When the strain exceeds $\gamma_{LVE}$, *G′* and *G″* decrease due to deformation of the chain-like structures in the ER fluid. The elastic stress (*τ′ = G′γ*) can be obtained using the strain amplitude sweep test experimental results. Figure 12b gives the elastic stress as a function of the *γ*. In the low strain amplitude region, the elastic stress value increased linearly with increasing *γ*, reaching a maximum value, which usually suggests structural breakdown and yield of the ER suspension. In addition, the correlation between the elastic yield stress and the electrical field strength is often the same as the dynamic yield stress and can be expressed by Equation (7), as shown in Figure 11d.

**Figure 13:**
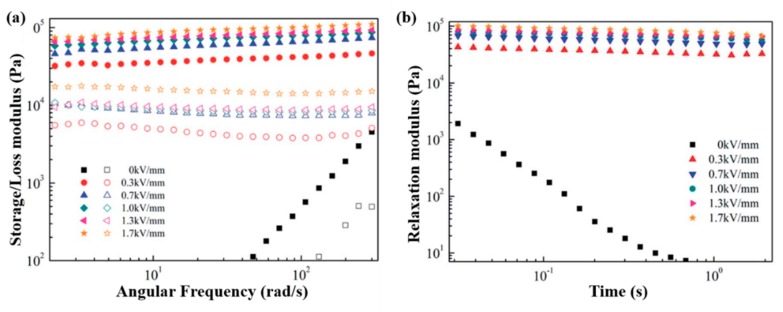
(**a**) Storage and loss modulus (closed symbol: *G′*, open symbol: *G″*) as a function of angular frequency and (**b**) relaxation modulus calculated by Schwarzl equation for the SiO_2_/PPy based ER fluid (10 vol %) (Kim et al. [95], © The Royal Society of Chemistry, 2016).

The angular frequency sweep tests are usually carried out with a constant *γ* within the $\gamma_{LVE}$. Figure 13a gives the angular frequency dependence of *G′* and *G″* for a SiO_2_/PPy-based ER suspension for different Es at a fixed strain of 0.004%. Without an electrical field, *G′* and *G″* show similar values and increase with increasing angular frequency. When an electric field is applied, *G′* is much higher than *G″* and shows a relatively stable value over the entire frequency range, suggesting that ER fluids become more solid-like with an input E. Moreover, the stress relaxation property can be estimated from a frequency sweep test. Figure 13b presents the relaxation modulus (*G*(*t*)) as a function of time. The *G*(*t*) was estimated from the measured *G′*(*ω*) and *G″*(*ω*) values in Figure 13a using the Schwarzl equation of Equation (8) [113]:

(8) $$G\left(t\right)\;≅{\;G}^{\prime }\left(\omega \right)\;–\;{0.566G}^{\prime \prime }\left(\omega /2\right)+{0.203G}^{\prime \prime }\left(\omega \right)$$

## 7. Magnetorheological Characteristics

### 7.1. Magnetic Particles

Magnetic particles used in MR fluids usually need to have soft magnetic properties, i.e., the amount of magnetization of the particles increases with increased external magnetic field strengths, and when the external magnetic field is turned off, the magnetism can be restored immediately to zero. Therefore, soft-magnetic carbonyl iron (CI) and magnetite (Fe_3_O_4_) have been studied as the most potential MR particles. Although CI has attracted significant attention because of its very high saturation magnetization, the density of CI is too high, which usually causes serious sedimentation problems, whereas Fe_3_O_4_ has a relatively low density and has a suitable saturation magnetization, so it is considered a promising MR candidate. This article focuses on the core-shell structured conducting polymer-coated nanoparticles in ER and MR fluids. Owing to the conducting shell, these materials were used mainly as ER materials. On the other hand, some composite materials with Fe_3_O_4_ as the core have also been assessed as MR materials, including Fe_3_O_4_/PANI [72], Fe_3_O_4_/PPy [97], and Fe_3_O_4_/POA [81]. By manufacturing a core-shell structured Fe_3_O_4_–conducting polymer composite, the composite material usually exhibits better sedimentation stability owing to its lower density than bare Fe_3_O_4_, and the polymer shell can protect the Fe_3_O_4_ particles from chemical corrosion or oxidation, giving more stable MR characteristics.

### 7.2. Magnetic Properties

**Figure 14:**
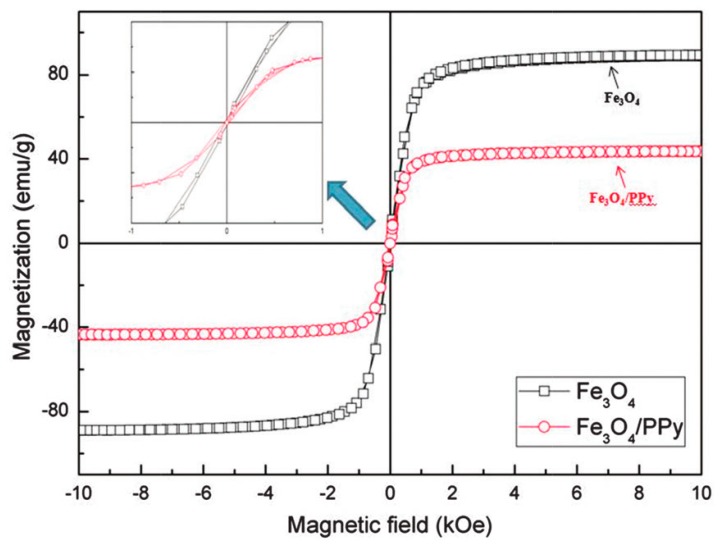
Magnetization curves of Fe_3_O_4_ and Fe_3_O_4_/PPy (Park et al. [97], © The Royal Society of Chemistry, 2015).

Similar to the response of the ER suspensions under electrical field stimulation, when the MR fluid is stimulated by an input magnetic field, the dispersed particles are arranged into chain structures in a direction parallel to the magnetic field because of the induced magnetostatic force, thereby causing the MR fluid to transform from a liquid-like state to a solid-like form. In addition, the MR characteristics of the MR fluid are closely related to the magnetic properties of the particles. Figure 14 shows the magnetization curves of Fe_3_O_4_ and Fe_3_O_4_/PPy tested using a vibrating sample magnetometer (VSM) with a magnetic field intensity ranging from −10 to 10 kOe. The saturation magnetization ($M_{s}$) of a material is an important parameter predicting the MR performance that can be achieved in a MR fluid. Generally, a larger MS value indicates higher MR performance of the MR fluid at the same concentration. As shown, the $M_{s}$ value of Fe_3_O_4_ and Fe_3_O_4_/PPy was 82 and 41 emu/g, respectively, indicating that the MR performance of Fe_3_O_4_/PPy-based MR fluid may be lower than that of Fe_3_O_4_. Furthermore, the hysteresis of magnetic particles has a direct influence on the recovery of an MR fluid upon the removal of the input magnetic field. When the material exhibits a large hysteresis loop, it indicates that the non-magnetic state of the material cannot be recovered immediately when the magnetic field is removed, which means that the magnetostatic interaction between the particles in an MR fluid cannot disappear over time and the chain structure cannot be eliminated. Hence, even if the magnetic field is removed, the MR fluid cannot immediately transition from a solid-like state to a fluid-like state. As shown in the inset in Figure 14, Fe_3_O_4_ and Fe_3_O_4_/PPy have almost no hysteresis loops, exhibiting soft magnetic properties, which mean that MR fluids based on them can sensitively transform between a solid-like and liquid-like state by applying and removing the magnetic field.

### 7.3. Steady Shear Tests

**Figure 15:**
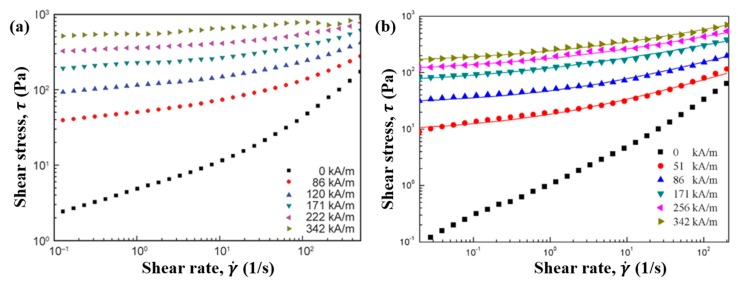
Shear stress curves of (**a**) Fe_3_O_4_/PANI based MR fluid (20 wt %) (Sim et al. [72], © BME-PT, 2015) and (**b**) Fe_3_O_4_/POA based MR fluid (10 vol %) (Lee et al. [81], © by authors, 2019).

Since the property of an MR suspension under a magnetic field has many similarities to that of an ER fluid under an electrical field, the main rheological characteristics of the MR fluid are introduced briefly below based on the preceding detailed description. Figure 15 shows the shear stress curves of Fe_3_O_4_/PANI and Fe_3_O_4_/POA-based MR fluids tested in CSR mode. Under an input magnetic field, the MR fluid exhibits Bingham fluid characteristics, and unlike the ER fluid, it maintains very stable τ in a low $\dot{\gamma }$ range. Therefore, in contrast to the CCJ equation used to explain the τ reduction phenomenon of ER fluids at a low $\dot{\gamma }$ range, the Herschel–Bulkley model is generally used to explain the τ curve of an MR fluid. The fitted lines in Figure 15b were generated from Herschel–Bulkley equation, which can be expressed as:

(9) $$\tau =\;\tau_{y}+K{\dot{\gamma }}^{n}$$

where K is defined as the consistency, and n is the index associated with the flow behavior.

**Figure 16:**
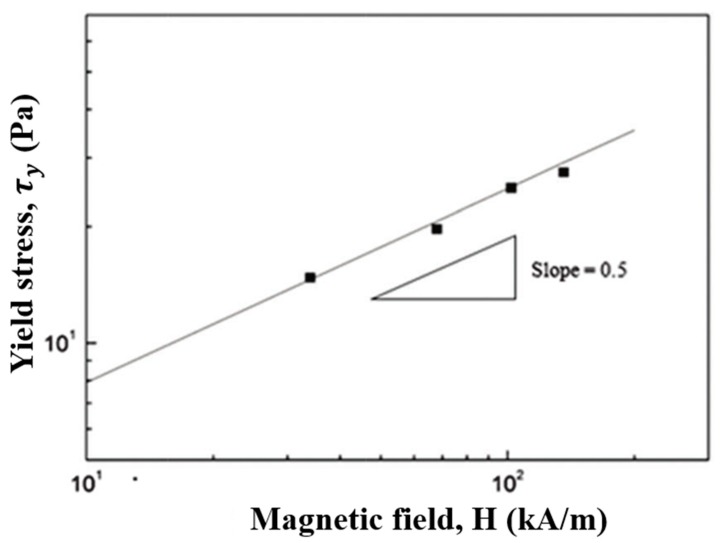
Dynamic yield stress of Fe_3_O_4_/PPy based MR fluid (10 vol %) as a function of magnetic field strength on a log-log scale (Park et al. [97], © The Royal Society of Chemistry, 2015).

The dynamic yield stress dependent on the magnetic field strength also can be expressed as:

(10) $$\tau_{y}\propto {\;H}^{\alpha }$$

where H is the magnetic field strength. On the other hand, because the non-magnetic polymer shell affects the magnetic properties of the magnetic core, the index α of an MR fluid based on the conducting polymer-covered magnetic particles occasionally exhibits a relatively low value. Figure 16 shows the dynamic yield stress of Fe_3_O_4_/PPy-based MR fluid as a function of the H on a log-log scale; the slope of the fitted line is 0.5.

### 7.4. Dynamic Oscillation Tests

**Figure 17:**
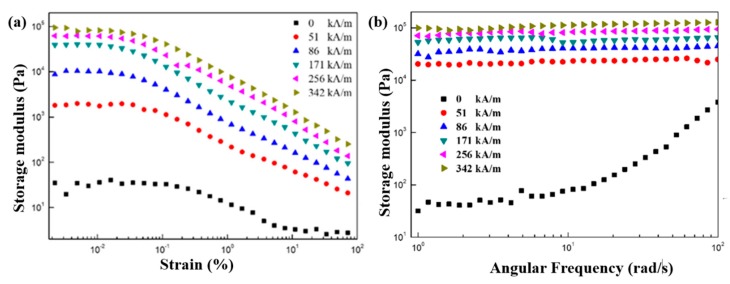
Dynamic oscillation tests of Fe_3_O_4_/POA based MR fluid (10 vol %): (**a**) storage modulus as a function of strain with fixed angular frequency of 6.28 rad/s; (**b**) storage modulus as a function of angular frequency with constant strain of 0.005% (Lee et al. [81], © by authors, 2019).

Figure 17 a shows the storage modulus (*G’*) of a Fe_3_O_4_/POA-based MR suspension as a function of strain under various Hs, and the fixed ω is 6.28 rad/s. When the H is applied, the value of *G’* increases by hundreds to tens of thousands of times and exhibits linear viscoelastic (*LVE*) behavior in a low strain range. In the high strain region, *G’* decreases significantly with increasing strain, showing that the chain-like structures induced by the external H are destroyed continuously. Therefore, when observing the frequency dependence of the *G’*, to avoid the damage to the MR fluid chain structure caused by excessive strain, the strain within the LVE region is usually selected as a fixed parameter. The *G’* value of the Fe_3_O_4_/POA-based MR fluid shows significant collapse at a strain of approximately 0.03%. Thus, the strain of 0.005% lower than the critical value was selected for the frequency sweep tests. Figure 17b shows the angular frequency dependence of $G^{\prime }$ with a constant strain of 0.005%. In the absence of the H, $G^{\prime }$ increased with increasing frequency, indicating that the MR fluid is in a fluid-like state at this time. On the other hand, when the H was applied, *G’* increased by several orders of magnitude and maintained a stable value throughout the frequency region. This means that, under the stimulation of the H, the particles in the MR fluid form stable chain-like structures, thereby transforming the MR fluid to a solid-like state.

## 8. Conclusions

This paper reviewed various core-shell structured conducting polymer-coated nanoparticles used in ER and MR fluids. Table 2 summarizes the parameters, including the synthesis method, morphology, size of nanoparticles used as a core, thickness of the conducting polymer shell, density ($\rho_{p}$), electrical conductivity ($\sigma_{p}$), electrical conductivity after de-doping, particle concentration of their based ER fluid, highest electric field strength applied ($E_{max}$), maximum dynamic yield stress (Max. $\tau_{dy}$), slope of the yield stress as a function of the electric field strength, and dielectric properties (Δε, λ).

In the case of conducting polymer-coated polymeric nanoparticles, PS and PMMA synthesized by a dispersion polymerization technique were employed. Chemical modification was usually required when the PMMA was used as a core, whereas, for the PS core, because of the presence of an aromatic ring structure, its combination with PANI and PANI derivatives can be carried out due to a π-π stacking interaction, which greatly simplifies the synthesis step. On the other hand, the inorganic cores include silica (SiO_2_), titanium dioxide (TiO_2_), ferric oxide (Fe_2_O_3_), magnetite (Fe_3_O_4_), and nanoclay. Since mineral oxides typically have hydroxyl groups on the surface, they are usually subjected to silanization to graft the corresponding functional groups on their surface before coating them with the conducting polymer. In addition, the clay shows a negative center in the present of acid, so that the monomer cations can be absorbed to the clay due to electrostatic interactions and then polymerize to form a polymer layer.

The yield stress under the electric field increases with increasing particle concentration in the ER fluid, but the morphology, size, and surface characteristics of the material have a significant influence on the rheological properties. Compared to spherical materials, rod or fibrous materials can usually contribute to higher ER efficiency, which may be because materials with a high aspect ratio can increase the contact area among the particles and induce a higher inter-particles frictional force. In the study of the ER fluid properties based on PANI/PSS-coated SiO_2_ and SiO_2_/PT with different sizes, the ER performance increases with decreasing particle size. In addition, a rough surface with a high specific surface area is also a favorable factor to contribute to high ER performance.

To prevent electrical breakdown of the ER fluids under an input electric field, the electrical conductivity of ER materials is generally less than 10^−6^ S/cm, and PANI-coated materials generally have electrical conductivities higher than 10^−3^ S/cm; hence, a de-doping process using an alkaline solution is necessary. On the other hand, the electrical conductivities of other conducting polymer-coated materials are relatively low, mainly when the thickness of the conducting polymer shell is very thin. Therefore, it is possible to have a suitable electrical conductivity without a de-doping step. Moreover, because the intrinsic conductivity of PDPA is relatively low (usually <10^−8^ S/cm), the PDPA coated material is expected to exhibit high safety when adopted in an ER fluid. If the electrical conductivity is too low, a wilting ER effect can occur. Therefore, a suitable conductivity of 10^−7^–10^−10^ S/cm as an ER material is recommended.

Furthermore, the sedimentation stability of the ER fluid has an important influence on its industrial applications. A common parameter for evaluating the sedimentation stability of ER fluids is the difference in density between the ER particles and carrier fluid. The table shows that the density of the polymer as a core is generally smaller than that when the inorganic substance is used as a core. Thus, the former is expected to exhibit better sedimentation stability. In addition to density, the morphology, size, and surface characteristics of the particles and particle concentration also have a striking effect on the stability of the precipitate. Overall, a high particle concentration can improve the sedimentation stability because the particle-particle interactions and particle-liquid interactions are enhanced at higher concentrations.

Cubic or rod-like particles have higher sedimentation stability than spherical particles, which may be due to the limited rotation of particles with cubic or rod-like structures in the carrier liquid. Moreover, the high specific surface area of particles can increase the inter-particle interaction, thereby reducing the sedimentation velocity. Even in materials with the same morphology, when the surface characteristics are different, the sedimentation stability also will be significantly different. For example, sea urchin-like PS/PANI showed better dispersion stability than PS/PANI with a smooth surface because of the more significant drag force and inter-particles frictional force from the high specific surface area of the sea urchin-like morphology. Moreover, the sedimentation stability also increased with decreasing particle size.

Therefore, the following characteristics should be satisfied when designing an ER material having both excellent ER characteristics and sedimentation stability: (1) a morphology with a high aspect ratio, such as cube, rod, tube, and fiber; (2) small size; (3) rough surface; (4) electrical conductivity between 10^−7^ and 10^−10^ S/cm; and (5) low density. Moreover, high particle concentration in the ER fluid can also promote high ER performance and excellent sedimentation stability.

In addition, the dielectric properties play an important role in predicting the ER performance of ER fluids. In general, a high *∆ε* value and a short *λ* can contribute to a more stable chain structure under an electric field to exhibit high ER performance. In some cases, however, the value of *λ* is short even when the value of *∆ε* is high, which is not always associated with a higher ER effect. Therefore, *∆ε* and *λ* cannot fully demonstrate the ER performance, and it is also necessary to consider the influence of other factors, such as the morphology, size, and surface characteristics of the particles.

Regarding the conducting polymer-coated nanoparticle-based MR fluid, the relevant parameters are summarized in Table 3. Fe_3_O_4_ is the most attractive core material because of its high saturation magnetization, soft magnetic properties, ease of synthesis, suitable particle morphology and size, and lower density than other magnetic materials.

The magnetic properties of the material are related directly to the MR properties of the MR fluid composed of it. The high saturation magnetization is expected to result in higher yield stresses of the MR fluid under magnetic fields, and the soft magnetic properties help increase the sensitivity of the MR fluid to transition between the solid and similar liquid states.

The most important function of coating the surface of a magnetic particle with a polymer is that a polymer shell with a small density can improve the sedimentation stability of the MR fluid significantly. As shown in Table 3, compared to bare Fe_3_O_4_-based MR fluid, the sedimentation stability of Fe_3_O_4_/PANI and Fe_3_O_4_/POA-based MR fluids are increased by approximately 45% and 50%, respectively. Moreover, the coated polymer can also protect the magnetic cores from corrosion and oxidation to improve the stability of the MR properties.

Furthermore, coating the magnetic particles with a conducting polymer can impart electrical conductivity to the composite to provide dual stimuli response characteristics in both electric and magnetic fields. The characteristics of Fe_3_O_4_/PANI and Fe_3_O_4_/PPy as both ER and MR materials have been studied. Therefore, conductive polymer-coated magnetic nanoparticles are expected to develop into new multifunctional materials.

On the other hand, there is a problem in that the coating with a conducting polymer inevitably affects the magnetic properties of the core. Hence, the development of a conducting polymer with a low density and low magnetic masking property will become a major challenge.

## Figures and Tables

**Table 1:** Fitting parameters of Equation (4) for ER fluids in Figure 8 (Eroal et al. [88], © The Royal Society of Chemistry, 2015).

ER Fluids (1.25 vol %)	$\varepsilon_{0}$	$\varepsilon_{\infty }$	Δε	λ (s)	α
Nanoparticle-TiO_2_	3.07	2.60	0.47	5 × 10^−3^	0.69
Nanoparticle-TiO_2_/PEDOT	3.20	2.60	0.60	3 × 10^−3^	0.63
Nanorod-TiO_2_	3.97	2.67	1.30	1.9 × 10^−5^	0.69
Nanorod-TiO_2_/PEDOT	4.20	2.52	1.68	1.3 × 10^−4^	0.72

**Table 2:** Parameters of conducting polymer-coated nanoparticles applied to ER fluids.

Material	Synthesis	Morphology	Core	Shell	$\rho_{p}\;(g/{\mathrm{cm}}^{3})$	$\sigma_{p}\;(S/\mathrm{cm})$	$\sigma_{p}\;\mathrm{after}\;\mathrm{De}-\mathrm{Doping}\;(S/\mathrm{cm})$	Concentration	$E_{\max}\;(\mathrm{kV}/\mathrm{mm})$	$\mathrm{Max}.\;\tau_{\mathrm{dy}}\;(\mathrm{Pa})$	Slope	Δ*ε*	*λ* (s)
PS/PANI [61]	Electrostatic interaction (sulfuric acid treatment)	Spherical	360 nm	70 nm	1.13	10^−3^	10^−8^	10 vol %	2.5	420	2.0	1.24	3 × 10^−5^
PS/PANI [66]	π-π stacking interaction	Sea urchin-like	1.0 μm	50 nm	1.29	1.13 × 10^−2^	2.24 × 10^−11^	10 vol %	3.0	102	1.5	2.27	5.7 × 10^−3^
SiO_2_/PANI [69]	π-π stacking interaction (aniline group modification)	Spherical	~1.0 μm	50 nm	1.87	1.8 × 10^−2^	5.8 × 10^−12^	20 vol %	3.0	187	2.0	0.98	1.5 × 10^−2^
PANI/PSS-coated SiO_2_ [70]	Seed-coating method (amino modification)	Spherical	50 nm	2 nm	2.57	_	_	5–30 vol %	3.0	~7200 (30 vol %)	2.0–1.5	_	_
			100 nm	2 nm	2.62	_	_	5–30 vol %	3.0	~5300 (30 vol %)	2.0–1.5	_	_
			250 nm	2 nm	2.64	_	_	5–30 vol %	3.0	~3200 (30 vol %)	2.0–1.5	_	_
Fe_3_O_4_/PANI [72]	Electrostatic interaction (HCl modification)	Spherical	0.7–1.0 μm	~100 nm	2.76	_	5.56 × 10^−8^	20 wt %	3.0	20	2.0	_	_
Fe_2_O_3_/PANI [73]	Surfactant (CTAB)	Flower-like	_	_	_	_	De-doping	20 wt %	3.0	~300	1.97	_	_
PANI/SPL [74]	Electrostatic interaction	Nano-needle	Length: 1–2.5 μm Diameter: 20-25 nm	_	_	_	De-doping	8 vol %	1.6	242	1.5	2.12	1.65 × 10^−4^
Pal/PANI [76]	Electrostatic interaction	Nano-fibrous	Length: ~μm Diameter: ~20 nm	_	1.77	10^−3^	10^−8^	10 vol %	2.5	512	2.0	2.04	2.1 × 10^−4^
PMMA-PNMA [77]	Chemical grafting (epoxy-amine reaction)	Spherical	700 nm	100 nm	_	9.319 × 10^−4^	5.35 × 10^−10^	10 vol %	3.5	212	1.5	_	_
PEAN-PMMA [78]	Chemical grafting (epoxy-amine reaction)	Spherical	700 nm	90 nm	_	_	without de-doping	10 vol %	3.5	458	2.0	0.89	9 × 10^−7^
SiO_2_/POA [80]	π-π stacking interaction (aniline group modification)	Spherical	~1.0 μm	50 nm	_	3.6 × 10^−7^	7.2 × 10^−10^	10 vol %	2.3	~100	1.5	3.9	1.7 × 10^−3^
PS/PEDOT [87]	Swelling-diffusion-interfacial polymerization	Spherical	1.05 μm	10 nm		10^−4^	10^−8^	10 vol %	1.8	13.4	1.5	_	_
TiO_2_/PEDOT [88]	Covalent bonding (amino modification-thiophene grafting)	Nano-rod	Length: ~μm Diameter: 30–100 nm	6–7 nm	2.253	2.69	7.8 × 10^−2^	1–5 vol %	3.0	640 (5 vol %)	_	1.68	1.3 × 10^−4^
		Spherical	_	_	2.558	2.63	6.8 × 10^−2^	1–5 vol %	3.0	15 (5 vol %)	_	0.6	3 × 10^−3^
PEDOT/SiO_2_ [89]	In situ polymerization without surfactant	Spherical -like	~300 nm	_	1.81	10^−6^	10^−8^	10 vol %	2.5	200	2.0	0.38	4 × 10^−6^
TiO_2_/P3OT [94]	Covalent bonding (amino modification-thiophene grafting)	Nano-cube	300–700 nm	12-30 nm	3.37	1.16 × 10^−5^	_	1.25–7.5 vol %	3.5	360 (7.5 vol %)	_	0.092	2.7 × 10^−4^
SiO_2_/PPy [95]	In situ polymerization (MPS modification)	Spherical	500 nm	90 nm	1.98	2.3 × 10^−8^	without de-doping	10 vol %	1.7	151	1.5	0.94	1.5 × 10^−4^
SiO_2_/PT [24]	Seeded polymerization (Oxidation treatment by Fe^3+^)	Spherical	7 nm	2 nm	_	1.0 × 10^−7^	without de-doping	5–30 vol %	3.0	3900 (30 vol %)	_	_	_
			11 nm	2 nm	_	1.0 × 10^−7^	without de-doping	5–30 vol %	3.0	~2300 (30 vol %)	_	_	_
			22 nm	2 nm	_	1.0 × 10^−7^	without de-doping	5–30 vol %	3.0	~1500 (30 vol %)	_	_	_
SiO_2_/PT [96]	Electrostatic interaction (Oxidation treatment by Fe^3+^)	Spherical	22 nm	2 nm	_	_	_	30 vol %	3.0	~1900	_	_	_
SiO_2_/PPy [96]			22 nm	2 nm	_	_	_	30 vol %	3.0	~2800	_	_	_
SiO_2_/PEDOT [96]			22 nm	2 nm	_	_	_	30 vol %	3.0	~3900	_	_	_
SiO_2_/PANI [96]			22 nm	2 nm	_	_	_	30 vol %	3.0	~4400	_	_	_
Fe_3_O_4_/PPy [97]	In situ polymerization (sonication assisted)	Spherical	456.6 nm	60 nm	2.24	10^−2^	10^−7^	10 vol %	0.8	~30	1.0	2.49	5 × 10^−3^
PS/PDPA [98]	π-π stacking interaction	Spherical	1.05 μm	16.12 nm	1.127	10^−11^	without de-doping	5 vol %	2.5	54.7	1.5	0.508	5.5 × 10^−4^

**Table 3:** Parameters of conducting polymer-coated nanoparticles applied to MR fluids.

Material	Synthesis	Morphology	Core	Shell	$\rho_{p}\;\;(g/{\mathrm{cm}}^{3})$	M_s_	Concentration	H_max_ (kA/m)	$\mathrm{Max}.\;\tau_{\mathrm{dy}}\;(\mathrm{Pa})$	Slope	Enhanced Sedimentation Ratio
Fe_3_O_4_/PANI [72]	Electrostatic interactions (HCl modification)	spherical	0.7–1.0 μm	~100 nm	2.76	38	20 wt %	342	437	2.0	~45%
Fe_3_O_4_/PPy [97]	In situ polymerization (sonication assisted)	spherical	456.6 nm	60 nm	2.24	41	10 vol %	136	~30	0.5	_
Fe_3_O_4_/POA [81]	Electrostatic interactions (HCl modification)	spherical	445 nm	60 nm	2.52	36	10 vol %	342	~200	1.5	50%

## References

[1] Gamerith C., Luschnig D., Ortner A., Pietrzik N., Guse J.-H., Burnet M., Haalboom M., van der Palen J., Heinzle A., Sigl E. (2019). pH-responsive materials for optical monitoring of wound status. Sens. Actuators B Chem..

[2] Abdollahi A., Roghani-Mamaqani H., Razavi B., Salami-Kalajahi M. (2019). The light-controlling of temperature-responsivity in stimuli-responsive polymers. Polym. Chem..

[3] Li M., Zhang H., Gao F., Tang Z., Zeng D., Pan Y., Su P., Ruan Y., Xu Y., Weng W. (2019). A cyclic cinnamate dimer mechanophore for multimodal stress responsive and mechanically adaptable polymeric materials. Polym. Chem..

[4] Martin J.E., Odinek J., Halsey T.C., Kamien R. (1998). Structure and dynamics of electrorheological fluids. Phys. Rev. E.

[5] Choi H.J., Jhon M.S. (2009). Electrorheology of polymers and nanocomposites. Soft Matter.

[6] Yin J., Zhao X. (2011). Electrorheology of nanofiber suspensions. Nanoscale Res. Lett..

[7] Sheng P., Wen W. (2010). Electrorheology: Statics and dynamics. Solid State Commun..

[8] Yamaguchi H., Zhang X.-R., Niu X.-D., Nishioka K. (2010). Investigation of impulse response of an ER fluid viscous damper. J. Intell. Mater. Syst. Struct..

[9] Lee I.S., Lee J.Y., Sung J.H., Choi H.J. (2005). Synthesis and electrorheological characteristics of polyaniline-titanium dioxide hybrid suspension. Synth. Met..

[10] Kim Y.D., Song I.C. (2002). Electrorheological and dielectric properties of polypyrrole dispersions. J. Mater. Sci..

[11] Seo Y.P., Seo Y. (2012). Modeling and analysis of electrorheological suspensions in shear flow. Langmuir.

[12] Ashour O., Rogers C.A., Kordonsky W. (1996). Magnetorheological fluids: Materials, characterization, and devices. J. Intell. Mater. Syst. Struct..

[13] Wang G., Zhao D., Li N., Zeng Y., Han S., Ma Y., Dong X., Yu R. (2019). Facile synthesis of hierarchically structured flower-like Fe_3_O_4_ microspheres for high-performance magnetorheological fluids. J. Ind. Eng. Chem..

[14] Bilyk V.A., Korobko E.V. (2015). Research of the influence of dissipative heating on the performance characteristics of electrorheological shock absorbers. J. Intell. Mater. Syst. Struct..

[15] Liu Y., Davidson R., Taylor P. (2005). Touch sensitive electrorheological fluid based tactile display. Smart Mater. Struct..

[16] Niu X., Liu L., Wen W., Sheng P. (2007). Microfluidic manipulation in lab-chips using electrorheological fluid. J. Intell. Mater. Syst. Struct..

[17] Nanthakumar A.J.D., Jancirani J. (2019). Design optimization of magnetorheological damper geometry using response surface method for achieving maximum yield stress. J. Mech. Sci. Technol..

[18] Keshav M., Chandramohan S. (2019). Geometric optimisation of magnetorheological valve using feedforward neural networks for distribution of magnetic flux density inside the valve. Smart Mater. Struct..

[19] Hwang Y.-H., Kang S.-R., Cha S.-W., Choi S.-B. (2019). A robot-assisted cutting surgery of human-like tissues using a haptic master operated by magnetorheological clutches and brakes. Smart Mater. Struct..

[20] Huo L., Liao F.-H., Li J.-R. (2011). Synthesis and electrorheological performance of nanosized composite with polar inorganic compounds. Compos. Sci. Technol..

[21] Plachy T., Sedlacik M., Pavlínek V., Stejskal J. (2015). The observation of a conductivity threshold on the electrorheological effect of p-phenylenediamine oxidized with p-benzoquinone. J. Mater. Chem. C.

[22] Stejskal J., Mrlík M., Plachý T., Trchová M., Kovářová J., Li Y. (2017). Molybdenum and tungsten disulfides surface-modified with a conducting polymer, polyaniline, for application in electrorheology. React. Funct. Polym..

[23] Kim D.-H., Kim Y.D. (2007). Electrorheological properties of polypyrrole and its composite ER fluids. J. Ind. Eng. Chem..

[24] Hong J.-Y., Kwon E., Jang J. (2009). Fabrication of silica/polythiophene core/shell nanospheres and their electrorheological fluid application. Soft Matter.

[25] Sim I.S., Kim J.W., Choi H.J., Kim C.A., Jhon M.S. (2001). Preparation and electrorheological characteristics of poly (p-phenylene)-based suspensions. Chem. Mater..

[26] Quadrat O., Stejskal J. (2006). Polyaniline in electrorheology. J. Ind. Eng. Chem..

[27] Gercek B., Yavuz M., Yilmaz H., Sari B., Unal H.I. (2007). Comparison of electrorheological properties of some polyaniline derivatives. Colloids Surf. A.

[28] Kim S.H., Choi H.J., Leong Y.-K. (2017). Flow curve analysis of a Pickering emulsion polymerized PEDOT:PSS/PS-based electrorheological fluid. Smart Mater. Struct..

[29] Oh S.Y., Kang T.J. (2014). Electrorheological response of inorganic-coated multi-wall carbon nanotubes with core–shell nanostructure. Soft Matter.

[30] Wang B., Zhao X. (2005). Core/shell nanocomposite based on the local polarization and its electrorheological behavior. Langmuir.

[31] Pavlínek V., Sáha P., Kitano T., Stejskal J., Quadrat O. (2005). The effect of polyaniline layer deposited on silica particles on electrorheological and dielectric properties of their silicone–oil suspensions. Physica A.

[32] Ji X., Zhang W., Jia W., Wang X., Tian Y., Deng L., Liu J. (2017). Cactus-like double-shell structured SiO_2_@ TiO_2_ microspheres: Fabrication, electrorheological performances and microwave absorption. J. Ind. Eng. Chem..

[33] Choi H.J., Zhang W.L., Kim S., Seo Y. (2014). Core-shell structured electro-and magneto-responsive materials: Fabrication and characteristics. Materials.

[34] Bellucci S., Bolesta I., Guidi M.C., Karbovnyk I., Lesivciv V., Micciulla F., Pastore R., Popov A., Velgosh S. (2007). Cadmium clusters in CdI2 layered crystals: The influence on the optical properties. J. Phys. Condens. Matter.

[35] Cao L., Park H., Dodbiba G., Fujita T. (2008). Dispersion of submicron Ni particles into liquid gallium. Magnetohydrodynamics.

[36] Lee J.Y., Kwon S.H., Choi H.J. (2019). Magnetorheological characteristics of carbonyl iron microparticles with different shapes. Korea Aust. Rheol. J..

[37] Seo Y.P., Kwak S., Choi H.J., Seo Y. (2016). Static yield stress of a magnetorheological fluid containing Pickering emulsion polymerized Fe_2_O_3_/polystyrene composite particles. J. Colloid Interface Sci..

[38] Abe H., Naka T., Sato K., Suzuki Y., Nakano M. (2019). Shape-controlled syntheses of magnetite microparticles and their magnetorheology. Int. J. Mol. Sci..

[39] Jiang W., Cao Z., Gu R., Ye X., Jiang C., Gong X. (2009). A simple route to synthesize ZnFe_2_O_4_ hollow spheres and their magnetorheological characteristics. Smart Mater. Struct..

[40] Kim M.W., Han W.J., Kim Y.H., Choi H.J. (2016). Effect of a hard magnetic particle additive on rheological characteristics of microspherical carbonyl iron-based magnetorheological fluid. Colloids Surf. A.

[41] Dong Y.Z., Choi H.J. (2018). Synthesis of organic–inorganic poly (diphenylamine)/magnetite composite particles and their magnetorheological response. IEEE Trans. Magn..

[42] Abshinova M.A., Kazantseva N.E., Sáha P., Sapurina I., Kovářová J., Stejskal J. (2008). The enhancement of the oxidation resistance of carbonyl iron by polyaniline coating and consequent changes in electromagnetic properties. Polym. Degrad. Stab..

[43] Cvek M., Mrlík M., Ilčíková M., Mosnáček J., Babayan V., Kuceková Z., Humpolíček P., Pavlínek V. (2015). The chemical stability and cytotoxicity of carbonyl iron particles grafted with poly(glycidyl methacrylate) and the magnetorheological activity of their suspensions. RSC Adv..

[44] Liu Y.D., Zhang K., Zhang W.L., Choi H.J. (2012). Conducting material-incorporated electrorheological fluids: Core-shell structured spheres. Aust. J. Chem..

[45] Cho Y.H., Cho M.S., Choi H.J., Jhon M.S. (2002). Electrorheological characterization of polyaniline-coated poly (methyl methacrylate) suspensions. Colloid Polym. Sci..

[46] Li Y., Wang Z., Wang C., Zhao Z., Xue G. (2011). Controlling the morphology of micrometre-size polystyrene/polyaniline composite particles by swelling-diffusion-interfacial-polymerization method. Polymer.

[47] Trlica J., Sáha P., Quadrat O., Stejskal J. (2000). Electrorheology of polyaniline-coated silica particles in silicone oil. J. Phys. D Appl. Phys..

[48] Fang Q., Zhang J., Bai L., Duan J., Xu H., Cham-Fai Leung K., Xuan S. (2019). In situ redox-oxidation polymerization for magnetic core-shell nanostructure with polydopamine-encapsulated-Au hybrid shell. J. Hazard. Mater..

[49] Xuan S., Wang Y.-X.J., Yu J.C., Leung K.C.-F. (2009). Preparation, Characterization, and catalytic activity of core/shell Fe_3_O_4_@polyaniline@Au nanocomposites. Langmuir.

[50] Zhang J., Fang Q., Duan J., Xu H., Xu H., Xuan S. (2018). Magnetically separable nanocatalyst with the Fe_3_O_4_ core and polydopamine-sandwiched Au nanocrystal Shell. Langmuir.

[51] Park I.H., Choi H.J. (2018). Fabrication of p-aminobenzoic acid grafted carbonyl iron/polyindole composite particles and their magnetorheological response. J. Ind. Eng. Chem..

[52] Min T.H., Choi H.J., Kim N.-H., Park K., You C.-Y. (2017). Effects of surface treatment on magnetic carbonyl iron/polyaniline microspheres and their magnetorheological study. Colloids Surf. A.

[53] Yang G., Takei T., Yanagida S., Kumada N. (2019). Hexagonal tungsten oxide-polyaniline hybrid electrodes for high-performance energy storage. Appl. Surf. Sci..

[54] Yang Y.-F., Meng F.-Y., Li X.-H., Wu N.-N., Deng Y.-H., Wei L.-Y., Zeng X.-P. (2019). Magnetic Graphene Oxide-Fe3O4-PANI Nanoparticle Adsorbed Platinum Drugs as Drug Delivery Systems for Cancer Therapy. J. Nanosci. Nanotechnol..

[55] Faisal M., Harraz F.A., Ismail A.A., Alsaiari M.A., Al-Sayari S.A., Al-Assiri M.S. (2019). Novel synthesis of Polyaniline/SrSnO_3_ nanocomposites with enhanced photocatalytic activity. Ceram. Int..

[56] Fang F.F., Dong Y.-Z., Choi H.J. (2018). Effect of oxidants on morphology of interfacial polymerized polyaniline nanofibers and their electrorheological response. Polymer.

[57] Sim B., Choi H.J. (2015). Facile synthesis of polyaniline nanotubes and their enhanced stimuli-response under electric fields. RSC Adv..

[58] Dong H., Prasad S., Nyame V., Jones W.E. (2004). Sub-micrometer conducting polyaniline tubes prepared from polymer fiber templates. Chem. Mater..

[59] Otsubo Y., Sekine M., Katayama S. (1991). Effect of surface modification of colloidal silica on the electrorheology of suspensions. J. Colloid Interface Sci..

[60] Voorn D., Ming W., Van Herk A. (2006). Clay platelets encapsulated inside latex particles. Macromolecules.

[61] Piao S.H., Gao C.Y., Choi H.J. (2017). Sulfonated polystyrene nanoparticles coated with conducting polyaniline and their electro-responsive suspension characteristics under electric fields. Polymer.

[62] Hiamtup P., Sirivat A., Jamieson A.M. (2006). Electrorheological properties of polyaniline suspensions: Field-induced liquid to solid transition and residual gel structure. J. Colloid Interface Sci..

[63] Yin J., Xia X., Xiang L., Qiao Y., Zhao X. (2009). The electrorheological effect of polyaniline nanofiber, nanoparticle and microparticle suspensions. Smart Mater. Struct..

[64] Marins J.A., Giulieri F., Soares B.G., Bossis G. (2013). Hybrid polyaniline-coated sepiolite nanofibers for electrorheological fluid applications. Synth. Met..

[65] Liu Y.D., Park B.J., Kim Y.H., Choi H.J. (2011). Smart monodisperse polystyrene/polyaniline core–shell structured hybrid microspheres fabricated by a controlled releasing technique and their electro-responsive characteristics. J. Mater. Chem..

[66] Kim D., Tian Y., Choi H.J. (2015). Seeded swelling polymerized sea urchin-like core–shell typed polystyrene/polyaniline particles and their electric stimuli-response. RSC Adv..

[67] Lee I.S., Cho M.S., Choi H.J. (2005). Preparation of polyaniline coated poly(methyl methacrylate) microsphere by graft polymerization and its electrorheology. Polymer.

[68] Liu Y.D., Fang F.F., Choi H.J. (2010). Core−shell structured semiconducting PMMA/polyaniline snowman-like anisotropic microparticles and their electrorheology. Langmuir.

[69] Park D.E., Choi H.J., Manh Vu C. (2016). Stimuli-responsive polyaniline coated silica microspheres and their electrorheology. Smart Mater. Struct..

[70] Lee S., Hong J.-Y., Jang J. (2013). Synthesis and electrical response of polyaniline/poly(styrene sulfonate)-coated silica spheres prepared by seed-coating method. J. Colloid Interface Sci..

[71] Wang B., Liu C., Yin Y., Yu S., Chen K., Liu P., Liang B. (2013). Double template assisting synthesized core–shell structured titania/polyaniline nanocomposite and its smart electrorheological response. Compos. Sci. Technol..

[72] Sim B., Chae H.S., Choi H.J. (2015). Fabrication of polyaniline coated iron oxide hybrid particles and their dual stimuli-response under electric and magnetic fields. Express Polym. Lett..

[73] Tian X., He K., Wang B., Yu S., Hao C., Chen K., Lei Q. (2016). Flower-like Fe_2_O_3_/polyaniline core/shell nanocomposite and its electroheological properties. Colloids Surf. A.

[74] Jang D.S., Choi H.J. (2015). Conducting polyaniline-wrapped sepiolite composite and its stimuli-response under applied electric fields. Colloids Surf. A.

[75] Liu Y., Liu P., Su Z. (2007). Core-shell attapulgite@polyaniline composite particles via in situ oxidative polymerization. Synth. Met..

[76] Chae H.S., Zhang W.L., Piao S.H., Choi H.J. (2015). Synthesized palygorskite/polyaniline nanocomposite particles by oxidative polymerization and their electrorheology. Appl. Clay Sci..

[77] Moon I.J., Kim H.Y., Choi H.J. (2015). Conducting poly(N-methylaniline)-coated cross-linked poly(methyl methacrylate) nanoparticle suspension and its steady shear response under electric fields. Colloids Surf. A.

[78] Kim H.Y., Choi H.J. (2014). Core–shell structured poly (2-ethylaniline) coated crosslinked poly (methyl methacrylate) nanoparticles by graft polymerization and their electrorheology. RSC Adv..

[79] Kwon S.H., Liu Y.D., Choi H.J. (2015). Monodisperse poly (2-methylaniline) coated polystyrene core–shell microspheres fabricated by controlled releasing process and their electrorheological stimuli-response under electric fields. J. Colloid Interface Sci..

[80] Lee C.J., Choi H.J. (2017). Fabrication of poly(o-anisidine) coated silica core–shell microspheres and their electrorheological response. Mater. Res. Express.

[81] Lee H.J., Lu Q., Lee Y.J., Choi J.H. (2019). Polymer-Magnetic Composite Particles of Fe_3_O_4_/Poly(o-anisidine) and Their Suspension Characteristics under Applied Magnetic Fields. Polymers.

[82] Krztoń-Maziopa A., Wyciślik H., Płocharski J. (2005). Study of electrorheological properties of poly(p-phenylene) dispersions. J. Rheol..

[83] Wei C., Zhu Y., Jin Y., Yang X., Li C. (2008). Fabrication and characterization of mesoporous TiO_2_/polypyrrole-based nanocomposite for electrorheological fluid. Mater. Res. Bull..

[84] Chotpattananont D., Sirivat A., Jamieson A.M. (2004). Electrorheological properties of perchloric acid-doped polythiophene suspensions. Colloid Polym. Sci..

[85] Cabuk M., Yavuz M., Unal H.I. (2016). Electrokinetic, electrorheological and viscoelastic properties of Polythiophene-graft-Chitosan copolymer particles. Colloids Surf. A.

[86] Corradi R., Armes S. (1997). Chemical synthesis of poly (3,4-ethylenedioxythiophene). Synth. Met..

[87] An J.S., Moon I.J., Kwon S.H., Choi H.J. (2017). Swelling-diffusion-interfacial polymerized core-shell typed polystyrene/poly(3,4-ethylenedioxythiophene) microspheres and their electro-responsive characteristics. Polymer.

[88] Erol O., Unal H. (2015). Core/shell-structured, covalently bonded TiO_2_/poly(3,4-ethylenedioxythiophene) dispersions and their electrorheological response: The effect of anisotropy. RSC Adv..

[89] Park D.E., Dong Y.Z., Choi H.J. (2018). Fabrication and electric stimuli-response of semiconducting poly(3,4-ethylenedioxythiophene)/silica nanocomposite particles. Eur. Polym. J..

[90] Sever E., Unal H.I. (2015). Colloidal properties of surface functionalized nanocube-TiO_2_/poly (3-octylthiophene) core/shell conducting nanocomposite. Appl. Surf. Sci..

[91] Groenendaal L., Jonas F., Freitag D., Pielartzik H., Reynolds J.R. (2000). Poly (3,4-ethylenedioxythiophene) and its derivatives: Past, present, and future. Adv. Mater..

[92] Liu Y.D., Kim J.E., Choi H.J. (2011). Core-shell structured monodisperse poly(3,4-Ethylenedioxythiophene)/poly(styrenesulfonic acid) coated polystyrene microspheres and their electrorheological response. Macromol. Rapid Commun..

[93] Kim S.H., Kim J.H., Choi H.J., Park J. (2015). Pickering emulsion polymerized poly (3,4-ethylenedioxythiophene): Poly (styrenesulfonate)/polystyrene composite particles and their electric stimuli-response. RSC Adv..

[94] Sever E., Unal H.I. (2018). Electrorheological, viscoelastic, and creep-recovery behaviors of covalently bonded nanocube-TiO_2_/Poly (3-octylthiophene) colloidal dispersions. Polym. Compos..

[95] Kim M.W., Moon I.J., Choi H.J., Seo Y. (2016). Facile fabrication of core/shell structured SiO_2_/polypyrrole nanoparticles with surface modification and their electrorheology. RSC Adv..

[96] Hong J.-Y., Jang J. (2010). A comparative study on electrorheological properties of various silica–conducting polymer core–shell nanospheres. Soft Matter.

[97] Park D.E., Chae H.S., Choi H.J., Maity A. (2015). Magnetite–polypyrrole core–shell structured microspheres and their dual stimuli-response under electric and magnetic fields. J. Mater. Chem. C.

[98] Kim M.H., Choi H.J. (2017). Core–shell structured semiconducting poly (diphenylamine)-coated polystyrene microspheres and their electrorheology. Polymer.

[99] Kim M.H., Bae D.H., Choi H.J., Seo Y. (2017). Synthesis of semiconducting poly (diphenylamine) particles and analysis of their electrorheological properties. Polymer.

[100] Dong Y.Z., Choi H.J. (2019). Electrorheological characteristics of poly (diphenylamine)/magnetite composite-based suspension. Materials.

[101] Fang F.F., Liu Y.D., Lee I.S., Choi H.J. (2011). Well controlled core/shell type polymeric microspheres coated with conducting polyaniline: Fabrication and electrorheology. RSC Adv..

[102] Zhang W.L., Piao S.H., Choi H.J. (2013). Facile and fast synthesis of polyaniline-coated poly(glycidyl methacrylate) core–shell microspheres and their electro-responsive characteristics. J. Colloid Interface Sci..

[103] Abdullah M.F., Hashim A.M. (2019). Improved coverage of rGO film on Si inverted pyramidal microstructures for enhancing the photovoltaic of rGO/Si heterojunction solar cell. Mater. Sci. Semicond. Process..

[104] Yin J., Xia X., Xiang L., Zhao X. (2010). Conductivity and polarization of carbonaceous nanotubes derived from polyaniline nanotubes and their electrorheology when dispersed in silicone oil. Carbon.

[105] Cabuk S., Unal H.I. (2015). Enhanced electrokinetic, dielectric and electrorheological properties of covalently bonded nanosphere-TiO_2_/polypyrrole nanocomposite. React. Funct. Polym..

[106] Shearer S.A., Hudson J.R. (2008). Fluid mechanics: stokes’ law and viscosity. Meas. Lab..

[107] Liu Y.D., Quan X., Hwang B., Kwon Y.K., Choi H.J. (2014). Core–shell-structured monodisperse copolymer/silica particle suspension and its electrorheological response. Langmuir.

[108] Tang X., Wu C., Conrad H. (1995). On the conductivity model for the electrorheological effect. J. Rheol..

[109] Seo Y. (2011). A new yield stress scaling function for electrorheological fluids. J. Non-Newton. Fluid Mech..

[110] Cho M., Choi H., Jhon M. (2005). Shear stress analysis of a semiconducting polymer based electrorheological fluid system. Polymer.

[111] Mallick K., Witcomb M.J., Dinsmore A., Scurrell M.S. (2005). Polymerization of aniline by auric acid: Formation of gold decorated polyaniline nanoballs. Macromol. Rapid Commun..

[112] Sim B., Bae D.H., Choi H.J., Choi K., Islam M.S., Kao N. (2016). Fabrication and stimuli response of rice husk-based microcrystalline cellulose particle suspension under electric fields. Cellulose.

[113] Emri I., Von Bernstorff B., Cvelbar R., Nikonov A. (2005). Re-examination of the approximate methods for interconversion between frequency-and time-dependent material functions. J. Non-Newton. Fluid Mech..

